# Electronic origins of photocatalytic activity in *d*^*0*^ metal organic frameworks

**DOI:** 10.1038/srep23676

**Published:** 2016-03-29

**Authors:** Maxim A. Nasalevich, Christopher H. Hendon, Jara G. Santaclara, Katrine Svane, Bart van der Linden, Sergey L. Veber, Matvey V. Fedin, Arjan J. Houtepen, Monique A. van der Veen, Freek Kapteijn, Aron Walsh, Jorge Gascon

**Affiliations:** 1 Catalysis Engineering, Department of Chemical Engineering, Delft University of Technology, Julianalaan 136, Delft, The Netherlands; 2 Department of Chemistry, University of Bath, Claverton Down, Bath BA2 7AY, UK; 3 Laboratory of Magnetic Resonance, International Tomography Center, Institutskaya 3A, Novosibirsk 630090, Russia; 4 Novosibirsk State University, Novosibirsk 630090, Russia; 5 Optoelectronic Materials, Department of Chemical Engineering, Delft University of Technology, Julianalaan 136, 2628 BL Delft, The Netherlands; 6 Department of Materials Science and Engineering, Yonsei University, Seoul, Korea

## Abstract

Metal-organic frameworks (MOFs) containing *d*^*0*^ metals such as NH_2_-MIL-125(Ti), NH_2_-UiO-66(Zr) and NH_2_-UiO-66(Hf) are among the most studied MOFs for photocatalytic applications. Despite structural similarities, we demonstrate that the electronic properties of these MOFs are markedly different. As revealed by quantum chemistry, EPR measurements and transient absorption spectroscopy, the highest occupied and lowest unoccupied orbitals of NH_2_-MIL-125(Ti) promote a long lived ligand-to-metal charge transfer upon photoexcitation, making this material suitable for photocatalytic applications. In contrast, in case of UiO materials, the *d*-orbitals of Zr and Hf, are too low in binding energy and thus cannot overlap with the π* orbital of the ligand, making both frontier orbitals localized at the organic linker. This electronic reconfiguration results in short exciton lifetimes and diminishes photocatalytic performance. These results highlight the importance of orbital contributions at the band edges and delineate future directions in the development of photo-active hybrid solids.

Metal-organic frameworks (MOFs) have attracted a great deal of interest during the last decades due to their unprecedented surface area, remarkable tuneability and the fascinating variety of possible combinations of constituting blocks. Several applications have been suggested, such as separation of gases^1^,^2^, catalysis^3^, drug delivery and sensing^4^. Photocatalysis was proposed in the early 2000 s inspired by an intuitive analogy between them and their corresponding oxides, often being semiconductors^5^,^6^. Despite the fact that the early reports of semiconducting properties of MOFs have been disputed^7^,^8^, this initial misconception led to the application of frameworks based on Zn, Ti and other transition metals in a variety of photocatalytic reactions such as oxidation of organic compounds^9^,^10^, reduction of metal ions^11^ and synthesis of solar fuels^12^. NH_2_-MIL-125(Ti) and NH_2_-UiO-66(Zr) clearly stand out as the most researched among the different MOF structures tested in photocatalysis to date. While these two MOFs are based on the same linker and crystallize in a rather similar topology, the metal ions constituting the inorganic nodes in the frameworks are different: Ti^4+^ forms octameric Ti_8_O_8_(OH)_4_ rings^13^ in NH_2_-MIL-125(Ti) and Zr^4+^ or Hf^4+^ form the M_6_O_4_(OH)_4_ clusters in the UiOs^14^.

Intuitively, the light absorption properties of MOFs are determined by the synergy between the organic ligand and the metal ion. In this case, the addition of the primary amine group to terephthalate alters the electronic properties and promotes absorption in the visible region of the solar spectrum^15^. It has been assumed that the three *d*^*0*^ metals (Ti, Zr and Hf) feature similar electronic properties, accepting electrons in their unoccupied *d* orbitals. Thus, one would expect that a photo-generated electron in this series of MOFs would result in ligand-to-metal charge transfer (LMCT). Although understanding the nature of the excited states and the redox levels of these MOFs is crucial for the rational design of new photo-active frameworks^16^, these important aspects still need to be clarified. The electronic origin of the photocatalytic activity is the focus of this manuscript.

LMCT is generally accepted in the MIL-125(Ti)-type materials and was clearly demonstrated by EPR^17^,^18^, flash photolysis^19^ and theory^20^. The highest occupied crystalline orbital (HOCO) of NH_2_-MIL-125(Ti) is localized at the organic linker and the lowest unoccupied crystalline orbital (LUCO) is centred at the Ti *d*-orbitals^21^. Furthermore, the photoexcitation process results in the formation of easily detectable coloured paramagnetic Ti^3+^ species.

The mechanism behind light-excitation in UiO-66(Zr) has been debated by several researchers. Matsuoka and co-workers reported the absence of both photocatalytic activity towards hydrogen evolution and EPR signatures of paramagnetic Zr^3+^ in NH_2_-UiO-66(Zr) attributed to the very negative redox potential of Zr^4+^-centred clusters^17^. In contrast, and in analogy to NH_2_-MIL-125(Ti) Li *et al*. and Wang *et al*. ascribed the lowest in energy absorption band to LMCT and reported EPR spectra for this Zr-based MOF as evidence^9^,^22^,^23^.

The LUCO positions determine the ability of these MOFs to perform photo-driven reductions such as H_2_ evolution and/or CO_2_ reduction. Walsh and co-workers calculated, in line with reported experimental results, that hydrogen evolution is indeed feasible by using X-MIL-125(Ti) because the LUCO potential is more negative than the redox potential of the normal hydrogen electrode (NHE)^24^. The analysis of the electronic density of states revealed that C, N and O contribute to the HOCO while LUCO is composed of Ti *d* and O *p*-orbitals, indeed in agreement with the EPR results.

This LUCO remains relatively unperturbed by the choice of linkers. Walsh *et al*. reported, based on simple Hammett argument^25^, that different substituents on the organic linker of MIL-125(Ti) can indeed reduce the band gap down to 1.3 eV for diaminoterephthalate^21^, yet this has little influence on the reductive potential of the excited MOF and is achieved *via* reducing the oxidative potential of the HOCO on the organic linker. In contrast, by changing the metal ions in the inorganic clusters^23^,^26^, the reducing potential of MIL-125(Ti) should be changed. For tuning the position of LUCO in NH_2_-UiO-66(X) the use of different *d*^*0*^-metals of the same group, Ti^4+^, Zr^4+^ and Hf^4+^ is an obvious choice. While MOFs based on these ions have photocatalyzed reductions yielding solar fuels, the effect of the different electronic structure has not been unravelled, nor has a comparison of their photocatalytic activity been reported. However, the fundamental understanding of how properties and activity of these photocatalytic MOFs relate to each other, has the potential to move forward this field greatly.

In this study we focus on the electronic properties of NH_2_-MIL-125(Ti), NH_2_-UiO-66(Zr) and NH_2_-UiO-66(Hf) and their influence on the photocatalytic performance in hydrogen evolution reaction (HER). For a fair comparison, the crystalline topology of these solids should be identical. These three structures were selected because of their clear similarities and the interest raised in the recent literature on their photocatalytic performance. In contrast to what has been recently reported, we demonstrate that only the Ti-based MOF exhibits appreciable catalytic activity while the frameworks of Zr and Hf are hardly active and exhibit nearly identical catalytic performance. Based on transient absorption spectroscopy, EPR and density functional theory (DFT), this difference is attributed to the nature of the excited states in these MOFs and emphasizes the importance of orbital contributions at the electronic ‘band’ edges.

## Results and Discussion

NH_2_-MIL-125(Ti) was synthesized by the protocol reported elsewhere^27^^SI^. The synthesis yielded a material with high crystallinity and total pore volume^7^ (Table S1, Fig. S3).

The UiO-type materials are known to possess structural defects, as demonstrated by the non-stoichiometric metal to linker ratios observed by several groups experimentally. Here we synthesized two types of NH_2_-UiO-66 that differ in the number of structural defects. They are denoted as X_d_ and X_i_ (X = Zr or Hf) and referred to as ‘defective’ and ‘ideal’, respectively. Based on the earlier works by Farha who reported the synthetic path yielding the defective UiO-66(Zr)^28^, Lillerud claiming the nearly stoichiometric composition of the UiO obtained with the high temperature synthesis^29^, and our own characterization, it can be safely assumed that the NH_2_-UiO-66 materials used possess a different number of structural defects^SI^. Zr_i_ has nearly a stoichiometric composition whereas Zr_d_ misses *ca.* 30% of linkers, in line with earlier observations by Farha *et al*.^28^.

**Figure 1:**
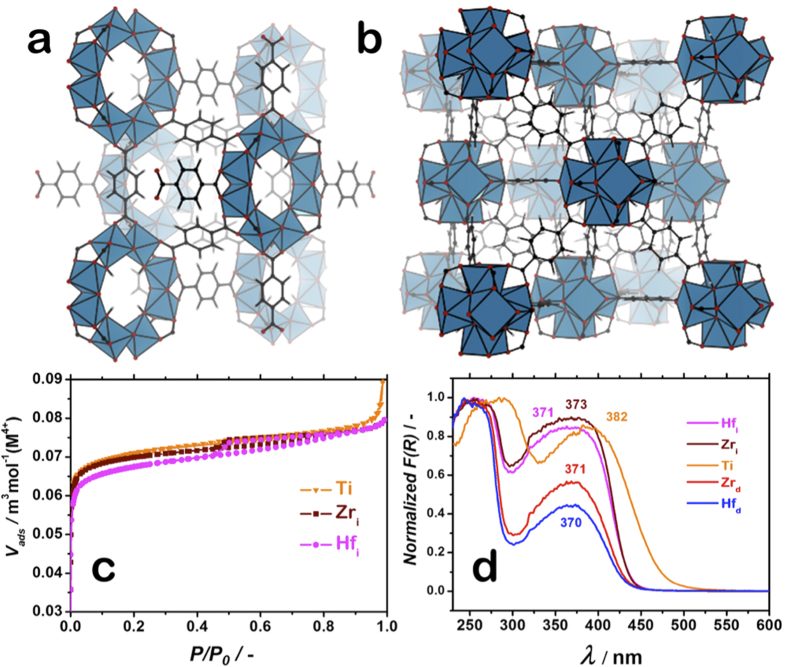
Structure and characterization of Metal-Organic Frameworks under investigation. Representation of the crystallographic structures of NH_2_-MIL-125(Ti) and NH_2_-UiO-66(X) (where X = Zr and Hf) have the same crystal structures as their parent non-aminated frameworks, shown in (**a**,**b**), respectively; Nitrogen physisorption isotherms for the catalysts used in this work normalized to the mass of metal constituting the MOFs (**c**); Diffuse reflectance UV-Vis spectra of catalysts studied in Kubelka-Munk representation (**d**); NH_2_-MIL-125(Ti) (*orange*), defective NH_2_-UiO-66(Zr) (*red*), ideal NH_2_-UiO-66(Zr) (*bordeaux*), defective NH_2_-UiO-66(Hf) (*blue*), ideal NH_2_-UiO-66(Hf) (*magenta*).

Figure 1c illustrates the N_2_ adsorption isotherms for Ti, Zr_i_ and Hf_i_ expressed per mol of the corresponding metal showing the similarity of the three structures, two of which of the same topology (UiO).

Optical properties of the synthesized Ti-, Zr–and Hf-based MOFs were investigated by diffuse reflectance UV-Vis spectroscopy (Fig. 1d). In line with the previous reports, NH_2_-MIL-125(Ti) possesses two absorption bands with the maxima at 280 and 382 nm^7^,^15^,^30^. The lowest in energy absorption band tails up to 475 nm, covering a fraction of the visible part of the electromagnetic spectrum. This transition is ascribed to LMCT. The spectra of Zr_i/d_ and Hf_i/d_ are blue shifted as compared to that of Ti, exhibiting a similar spectral profile with two distinct absorption bands *λ*_max_ = 255 and 370 nm. Li and co-workers attributed the 370 nm absorption band to LMCT, analogously to the case of NH_2_-MIL-125(Ti)^22^. They ascribed this absorption band to an *n*-π* transition of the linker and this excitation is followed by electron injection from a π* orbital to the zirconium core, in contrast to the claims of Matsuoka^17^.

The experimental HOCO-LUCO gap for NH_2_-MIL-125 is 2.75 eV (Fig. S6), in good agreement with previous reports^7^,^21^,^30^. On the other hand, the HOCO-LUCO gaps of Zr_i_ and Hf_i_ are identical and equal to 2.92 eV. Zr_d_ is at 2.95 eV and Hf_d_ is at 2.97 eV. While the HOCO-LUCO gaps of photocatalysts define the operation regime *i.e.* wavelength of light that can be utilized for chemical transformations, the absolute positions of HOCO and LUCO determine the thermodynamic feasibility of certain oxidations and reductions. To the best of our knowledge, the ionization potentials of NH_2_-MIL-125(Ti) and NH_2_-UiO-66(Zr or Hf) have not been reported to date.

**Figure 2:**
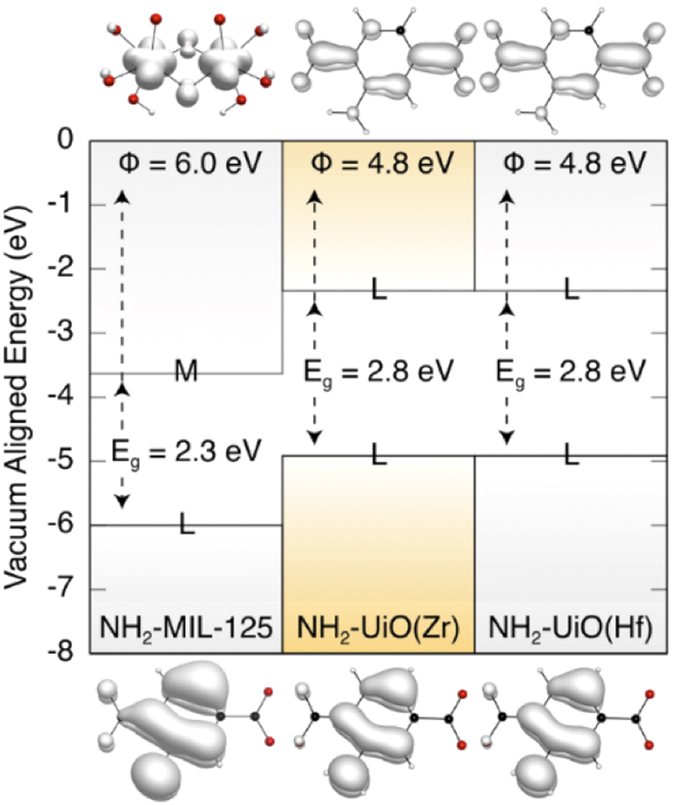
Electronic band alignment relative to the vacuum level of the three MOFs considered here from DFT calculations. Shown above and below are the orbitals contributing to the HOCO (*lower*) and LUCO (*upper*). M and L correspond to the localization of these orbitals on metal or ligand, respectively.

Estimation of the heterogeneous H_2_ redox potential is challenging, but applying the values determined in the solvated phase, we know this energy is near the vacuum level (*ca.* 4.4 eV). Considering only the energies of the frontier bands of the heterogeneous catalyst is not enough to predict the efficiency and activity of the material in the H_2_ evolution reaction. From a purely thermodynamic perspective, the lower the electron affinity the greater the HER turnover should be. Thus, considering our computational findings^SI^ depicted in Fig. 2, we would anticipate that the Zr–and Hf–MOFs should perform better than the Ti–material. However, we must also consider the identity of the orbitals that contribute to the frontier bands (LUCO).

Indeed, Fig. 2 shows that the LUCO for the MIL-125 structure is centred on the TiO_2_ (inorganic) region, whilst the two UiO-materials have a different electronic structure. Both the Zr-and Hf-materials have frontier band extrema located at the organics. This difference in the LUCO structure originates from the fact that the empty *d*-orbitals of Zr and Hf are too low in binding energy (close to the vacuum level) and thus do not overlap with the π* orbital of the ligand despite the suitability of the geometrical arrangement of the orbitals. At the same time the *d*-orbitals of Ti fulfil both the spatial and energetic requirements, allowing for an efficient overlap. The DFT electronic structure infers that the single electron excited state of NH_2_-MIL-125 should result in the formation of a delocalised Ti^3+^ species (which can localise by protonating a bridging oxide)^20^, while no corresponding M^3+^ can be formed in the UiO structures. In the UiO series of materials, the organic-centred excitons are expected to be short-lived, although still some catalytic activity could be expected, since the thermodynamic energy level alignment is in favour of reducing H_2_^31^.

**Figure 3:**
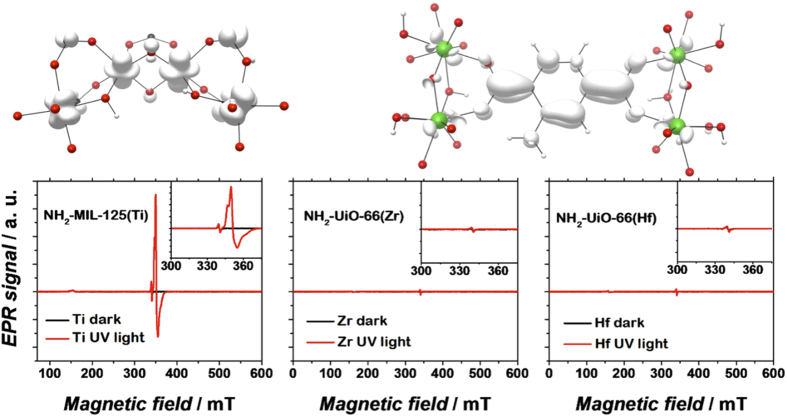
EPR spectra of dark (*black*) and UV-illuminated (*red*) MOFs: NH_2_-MIL-125(Ti) (*left*), ‘ideal’ NH_2_-UiO-66(Zr) (*centre*) and NH_2_-UiO-66(Hf) (*right*). The photoexcited electron is primarily Ti-centred in the case of NH_2_-MIL-125, whereas in the UiO-type materials, the electron resides on the organic linker.

In order to investigate the nature of excited states in the photocatalytic metal-organic frameworks we carried out electron paramagnetic resonance spectroscopy (EPR), shown in Fig. 3. The MOFs were subjected to conditions similar to those during photocatalytic reaction as described in the experimental part. In line with the previous reports^17^,^18^, illuminating NH_2_-MIL-125(Ti) with an energy corresponding to the HOCO-LUCO transition leads to the appearance of an intense paramagnetic signal ascribed to Ti^3+ ^13,17,18. Most importantly, NH_2_-UiO-66(Zr) and NH_2_-UiO-66(Hf) exposed to the same conditions did not develop any additional paramagnetic features upon illumination. This result is in agreement with the data reported by Matsuoka and colleagues^17^. Most likely, if any paramagnetic species evolve as a result of irradiating the NH_2_-UiO-66(X) solids, their lifetimes are too short for detection in the chosen experimental fashion.

So in principle, all MOFs subjected to the investigations have LUCO positions suitable to drive hydrogen evolution. In fact, the computational findings infer that Zr and Hf, when excited, possess an even greater thermodynamic driving force for accomplishing the desired reduction due to a lower electron affinity. However, reduction of the metal node only takes place in case of the Ti MOF.

**Figure 4:**
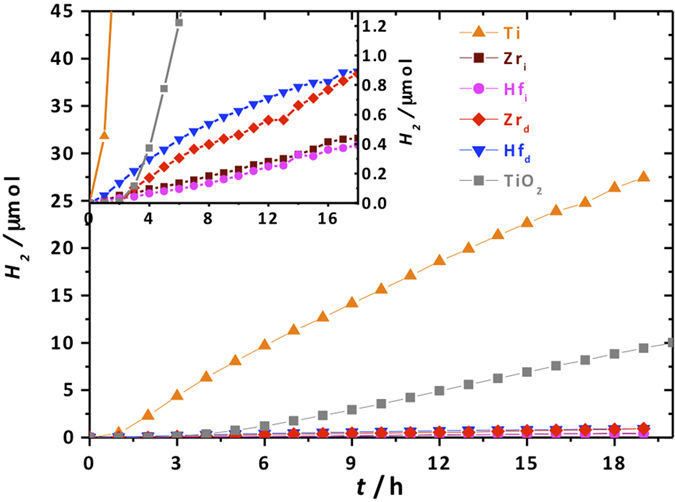
Photocatalytic performance of the MOF catalysts in hydrogen evolution: NH_2_-MIL-125(Ti) (*orange*), defective NH_2_-UiO-66(Zr) (*red*), ideal NH_2_-UiO-66(Zr) (*bordeaux*), defective NH_2_-UiO-66(Hf) (*blue*), ideal NH_2_-UiO-66(Hf) (*magenta*), Degussa P25 (*grey*). The inset magnifies the region of interest for the UiO-type catalysts.

The photocatalytic activity of the frameworks was assessed in visible light-driven hydrogen evolution. NH_2_-MIL-125(Ti) clearly outperforms defective NH_2_-UiO-66(Zr) and NH_2_-UiO-66(Hf) (Fig. 4). The latter two have practically the same photocatalytic activity that is *ca.* 30 times lower than that of Ti. The rates of hydrogen evolution under visible light were determined by a linear fit of the hydrogen produced *vs*. time and found to be 49.3, 1, 1, 1.7 and 1.7 μmol·h^−1^·g_catalyst_ for Ti, Zr_i_, Hf_i_, Zr_d_ and Hf_d_, respectively. Titanium dioxide Degussa P25 was used as a benchmark and exhibited hydrogen evolution rate of 18.3 μmol·h^−1^·g_catalyst_ at the same reaction conditions. Stability of NH_2_-MIL-125(Ti)^17^,^32^, NH_2_-UiO-66(Zr) catalysts and their analogues^23^ has been studied before and thus taken out of consideration. Interestingly, the defective samples exhibit an activity twice as high as the ideal ones and outperform them even when corrected for the amount of the corresponding metal (Table 1).

This difference in photocatalytic performance may originate from (i) a substantially smaller particle size of the defective solids and thus a larger external surface; (ii) a larger BET area; (iii) a positive influence of the defects on the kinetics of photocatalysis.

The first reasoning is particularly important in the case of dense oxides where chemical reactions can only occur at the surface of photocatalyst particles. However, we expect that in the case of MOFs this argument is of minor importance unless bulky substrates are subjected to photocatalysis. At first sight the second scenario seems the most logical: the BET area of the defective catalysts is much larger than the one of ideal MOFs. While this is true for Zr_d_ vs. Zr_i_, the case of NH_2_-UiO-66(Hf) suggests that the main contribution to the catalytic performance is not the difference in surface areas. The BET areas of Hf_d_ and Hf_i_ are 789 and 706 m^2^ g^−1^ respectively. Such small difference should not account for doubling the catalytic rate. The difference in optical properties is also negligible when speaking of such rate enhancement. In fact, the external quantum efficiencies of defective solids are twice as high as the ones of ideal MOFs as summarized in Table 1. This suggests that the structural defects within the UiO-type solids improve the hydrogen evolution rate^33^.

As already anticipated, the observed difference in activity between the Ti and the Hf, Zr catalysts can only be explained by the location of the LUCO at the inorganic node in case of NH_2_-MIL-125(Ti). Indeed, even taking into account the difference in optical properties, NH_2_-MIL-125(Ti) achieves an external quantum efficiency (EQE) *ca.* 13 times larger than the one of defective NH_2_-UiO-66(Zr) (Table 1). In order to further prove this hypothesis, transient absorption spectroscopy (TAS) was applied to study the lifetime of the excited states in these solids.

**Figure 5:**
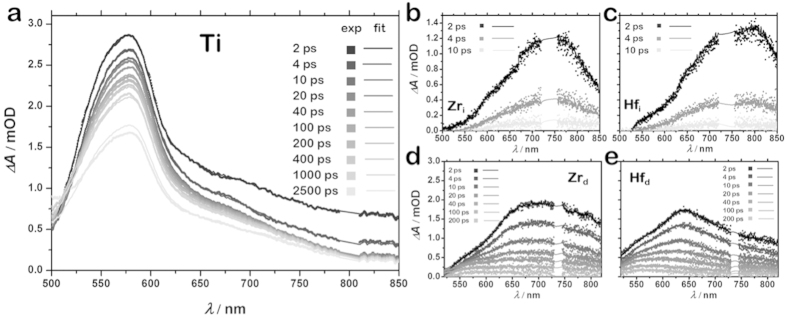
Transient absorption spectroscopy. Differential transient absorption spectra for NH_2_-MIL-125(Ti) upon excitation at 400 nm (**a**); Differential transient absorption spectra for ‘ideal’ (**b**) and ‘defective’ (**d**) NH_2_-UiO-66(Zr) and ‘ideal’ (**c**) and ‘defective’ (**e**) NH_2_-UiO-66(Hf) upon excitation at 370 nm. Symbols represent experimental data and lines the fits.

Results are presented in Fig. 5. The MOFs were excited at the wavelengths correspond corresponding to their respective absorption maxima. Excitation of NH_2_-MIL-125(Ti) results in an intense transient signal with a maximum at 560 and 600 nm, and a very broad peak from <500 nm till over 860 nm. A transient kinetic model with three consecutively decaying states was necessary to obtain a reasonable fit for the experimental data^34^^SI^. The third transient state has a moderately long lifetime of up to 9 ns (Table 2).

The transient spectra of NH_2_-UiO-66(Zr) and NH_2_-UiO-66(Hf) are markedly different from the one of Ti. The transient signals of Zr_i_ and Hf_i_ are nearly identical in terms of both decay kinetics and the spectral signatures^SI^. Right after the laser pulse, a broad transient signal emerges at 750 nm and decays rapidly to the ground state. The lifetime of this signal is 1.5 ps in both cases.

The defective solids exhibit a different behaviour. First of all, a transient kinetic model with three consecutive states, after which the material decays to the ground state, was necessary to obtain a reasonable fit for the experimental data (see SI)^34^. Moreover, the transient absorption band is shifted to shorter wavelengths compared to the ones of Zr_i_ and Hf_i_. The excited states of Hf_d_ and Zr_d_ have significantly longer lifetimes up to 190 and 130 ps, respectively. So, these samples have distinct properties different from Zr_i_ and Hf_i_. In addition to the rapid decay pathway registered in the case of ideal crystals, the defective Zr_d_ and Hf_d_ also undergo a relaxation pathway with longer lifetimes, although still two orders of magnitude shorter than the ones of Ti (Table 2).

Based on the EPR data and the computational results, the transient signals in the case of Hf and Zr structures can be assigned to the HOCO-LUCO transition of the NH_2_-UiO-66(X) framework. This transition is purely ligand-based and is independent of the metal ion employed. In contrast, in the Ti MOF, as established by EPR, Ti^3+^ is generated upon illumination. The main difference between the Zr/Hf and the Ti transient spectra, is the occurrence of absorption bands at much lower wavelengths in NH_2_-MIL-125(Ti), namely 560 and 600 nm, assigned to the stabilisation of the hole over the organic linker35, in contrast to earlier works where absorbance of similar materials with photo-generated Ti^3+^ occurs in the 500–600 nm range and is usually assigned to such Ti^3+^ species^36^,^37^.

These results are in line with the photocatalytic experiments. Indeed, a more efficient photocatalysis is expected when photo-excited charges have a longer lifetime. The longer lifetime for the NH_2_-MIL-125(Ti) with respect to the NH_2_-UiO-66(Zr) and NH_2_-UiO-66(Hf) is also in accordance with the EPR and DFT results. For MOFs where the lowest energy transition resides purely at the organic linker, as opposed to a LMCT, the lifetime of excited states is too short as to allow for efficient utilization of the photogenerated electrons. Such long-lived LMCT can be achieved when the energy of metal orbitals is close to the one of the π*-orbital of the organic linker thus allowing for an efficient overlap. In the case of aminoterephthalate, Ti appears to be an excellent metal. However, even metal ions with lower binding energies can potentially be utilized in this fashion as long as the appropriate linker is concerned.

## Conclusions

The photocatalytic potential of the group IV *d*^*0*^ metal-based metal-organic frameworks NH_2_-MIL-125(Ti), NH_2_-UiO-66(Zr) and NH_2_-UiO-66(Hf) was explored. First-principles electronic structure theory indicates that each of these MOFs has a LUCO position suitable for driving the hydrogen evolution reaction. However, experimentally the visible light photocatalytic activity of NH_2_-MIL-125(Ti) is much higher than the UiO MOFs. This remarkable difference can be understood in terms of the kinetics of the corresponding photoexcited states. While the LMCT excited state of NH_2_-MIL-125(Ti) is stabilized by the formation of Ti^3+^, excitation of NH_2_-UiO-66(Zr) and NH_2_-UiO-66(Hf) does not promote the formation of corresponding M^3+^ as is evident from the DFT and EPR studies. In contrast to NH_2_-MIL-125(Ti), the HOCO-LUCO transitions of NH_2_-UiO-66(X) are purely ligand-based and have a short lifetime accounting for the poor catalytic performance. The *d*-orbitals of Zr and Hf are too close to the vacuum level (low electron affinity) and do not overlap with the π* orbital of the ligand despite their geometrical suitability. Structural defects of the UiO MOFs result in twice higher catalytic rates due to the longer lifetime of the photoexcited states.

This work signifies the importance of the identity of orbitals defining the band extrema. The LMCT excited state of NH_2_-MIL-125(Ti) has a superior lifetime as compared to the organic-based states of the UiOs. The design of MOF for photocatalysis should focus on sufficiently long excited state lifetimes, thus improving catalytic activity.

## Experimental

### Materials and reagents

All chemicals were purchased from Sigma Aldrich and used without further purification. Methanol and dimethylformamide (DMF) were additionally dried over molecular sieve (zeolite 5 Å).

### Syntheses

#### NH_2_-MIL-125(Ti)

The MOF was synthesized using the protocol reported by Moreira and co-workers^27^. In a typical synthesis, 2.86 g (15.8 mmol) of 2-aminoterephthalic acid was dissolved in a mixture of 40 mL dry N, N-dimethylformamide (DMF) and 10 mL dry methanol (at room temperature). Then 2.86 mL (9.7 mmol) of titanium isopropoxide were added and the mixture was placed in an autoclave. The autoclave was sealed and the mixture was heated for 72 h at 110 °C. The obtained yellow solid was filtered, dispersed in fresh DMF and kept under stirring overnight (50 mL DMF per 1 g product) in order to remove residual linker. Then, the same procedure was repeated twice using methanol instead of DMF to exchange the DMF within the pores. The solid was finally dried under air at 100 °C.

#### NH_2_-UiO-66(Zr/Hf) ‘defective’

The protocol is based on the procedure reported by Farha and co-workers^28^. For the synthesis of NH_2_-UiO-66(X), where X = Zr or Hf, ZrCl_4_ (250 mg) or HfCl_4_ (346 mg) were dissolved in DMF (10 mL) in the presence of concentrated HCl (2 mL) upon sonication. Additional DMF (15 mL) and 2-aminoterephthalic acid (268 mg) were introduced and the mixture was placed in an autoclave and heated overnight at 80 ^o^C. The pale yellow solids were filtered off, suspended in DMF and sonicated for 10 min. This procedure was repeated twice with DMF and then twice with methanol. The solid was dried under air at 100 °C.

#### NH_2_-UiO-66(Zr/Hf) ‘ideal’

These MOFs were synthesized following the protocol reported by Lillerud *et al*.^29^ ZrCl_4_ (945 mg) or HfCl_4_ (1.308 g) were dissolved in DMF (24.4 mL) in the presence of concentrated HCl (0.7 mL) upon sonication. 1.467 g of 2-aminoterephthalic acid were introduced and the mixture was then heated for 20 h at 220 ^o^C in an autoclave. The pale yellow precipitate was filtered off and washed twice with DMF at 100 ^o^C (10 h each), then methanol at 100 ^o^C (10 h each).

### Characterization

*Powder X-Ray diffraction* patterns were recorded using Bruker-AXS D5005 with Co-*Kα* radiation.

*N*_*2*_*-physisorption* experiments were carried out at 77 K in a TriStar II unit gas adsorption analyser (Micromeritics). Prior to the measurements the samples were degassed at 423 K under vacuum for 16 h. The BET areas were calculated using intervals allowing positive BET constants^38^. The total pore volumes were calculated at 0.9 relative pressure.

*Scanning electron microscopy* (SEM) was carried out using a JEOL JSM-6010LA InTouchScope microscope.

*Thermogravimetric analysis* was performed by means of Mettler Toledo TGA/SDTA851e, under an air flow of 60 ml min^−1^ at a heating rate of 10 K min^−1^ up to 1073 K.

*Diffuse reflectance UV/Vis* spectra were collected using a Perkin–Elmer Lambda 900 spectrophotometer equipped with an integrating sphere (“Labsphere”) in the 200–800 nm range. BaSO_4_ was used as a white standard.

#### EPR spectroscopy

Steady-state EPR measurements were carried out at X-band (9.52 GHz) using a commercial EPR spectrometer Bruker Elexsys E580 equipped with an Oxford Instruments temperature control system (*T* = 4 –300 K). All spectra were acquired at 40 K. Samples were prepared by suspending 25 mg MOF in a solution of electron donor triethylamine (TEA)/CH_3_CN/H_2_O = 1/5/0.1 (total volume 200 μL). The volume of suspension placed into the EPR resonator was 50–60 μL. Each sample was degassed by several freeze-pump-thaw procedures and then sealed in an EPR quartz tube (OD 3.8 mm, ID 2.8 mm). When needed, the samples were exposed to a 500 W mercury lamp equipped with an IR filter (H_2_O, 7 cm optical path) and a UFS6 filter (see Fig. S7) for 30 min. After this period the sample tube was placed into liquid nitrogen. The cool-down time to frozen state was ~ 10–15 s. Then it was inserted into EPR cryostat.

#### Femtosecond Transient Absorption Spectroscopy

Samples were excited using 180 fs pulses at 400 nm for NH_2_-MIL-125(Ti) and 370 nm for NH_2_-UiO-66(Zr) and NH_2_-UiO-66(Hf) generated in a YKGBW oscillator (Light Conversion, Pharos SP) at 1028 nm through nonlinear frequency mixing in an OPA and second harmonics module (Light Conversion, Orpheus). A small fraction of the 1028 nm fundamental beam was split off to generate the broadband probe spectrum in a sapphire (500–1600 nm) crystal. The probe pulse was delayed relative to the pump using a delay stage with maximum delay of 3 ns. The pump and probe pulses overlap on the sample position under an angle of ~8 degrees, after which the probe light is led to a detector suitable for the probe spectrum selected (Ultrafast Systems, Helios). In order to prevent multiple photons absorption processes, the pump fluence was set sufficiently low, allowing us to study single exciton dynamics. In a typical experiment 2.7 mg MOF was dispersed in acetonitrile (700 μL) and sonicated for 30 min. In order to separate large particles (>100 nm), the suspension was then centrifuged for 8 min at 10000 rpm. The supernatant was placed in a 2 mm stirred quartz cuvette for the measurements. Transient data were analyzed using a global fitting routine in which the spectral evolution of the time-dependent absorption difference spectra is fitted to a sequential model yielding evolution-associated difference spectra (EADS)^39^. In the case of the NH_2_-MIL-125(Ti) and NH_2_-UiO-66(Zr/Hf) ‘defective’ a three-state kinetic model was used to fit the experimental data^34^. An additional state was used to force the system to decay to the ground state. All time traces were satisfactorily fitted with three time constants. The last state decays to the ground state being part of the model. For the NH_2_-UiO-66(Zr/Hf) ‘ideal’ one excited state (which population’s decay is defined by exp(−*kτ*)) that goes to the ground state (described by {1 − exp(*−kτ*)}) was sufficient for a good fit.

### Computational Methods

All calculations were performed within the framework of density functional theory with electron exchange and correlation treated with the semi-local PBEsol functional (for structure relaxation) and the non-local screened HSE06 functional (for quantitative electronic structure information). The crystalline MOFs were described within periodic boundary conditions are implemented in the VASP computational chemistry package^40^. A 500 eV planewave cut-off and Gamma point sampling of the first Brillouin zone was found to be sufficient for energy convergence within 0.01 eV per atom. Starting with experimentally collected crystallographic structures, lattice parameters and atomic positions were relaxed with PBEsol to equilibrium structures, which are available in an external data repository: https://github.com/WMD-Bath/Crystal_Structures. The optimized structures were then computed with HSE06 to recover the electronic structure. Electron energies were aligned to the vacuum level using a procedure developed by Butler, Hendon and Walsh, and the code is freely available^24^. A model of the triplet state (*i.e.* a first excited state) was obtained by fixing the total spin moment in the crystallographic unit cell (NUPDOWN = 2 in VASP). Images were made in VESTA^41^.

### Photocatalytic studies

Photocatalytic experiments were carried out using a home-built set-up equipped with a 500 W Xe/Hg lamp (66983, Newport). It consists of a custom-made Pyrex-glass reactor, a CP 9001 gas chromatograph (Chrompack) for analysis of the headspace, a KSLA gas pump and the light source. Light intensity was measured with AvaSpec-3648-2-USB2 (Avantes, the Netherlands). The reactor has a volume of 42.1 mL and is equipped with a water jacket to allow for precise temperature control. The light emitted by the lamp passes through a lens assembly (77330, Newport) focusing the beam on the reactor window, an H_2_O filter (61945, Newport) and a 385 nm cut-off optical filter. The pump is applied to ensure a sufficient mixing of gases in the headspace of the reactor and the stainless steel tubes (2.5 mL/min continuous operation). Every 60 min a probe of the headspace is analyzed by the GC. In a typical experiment 30 mg of a MOF were suspended in 23.5 mL of CH_3_CN, 4.7 mL of TEA and 0.5 mL of H_2_O. The suspension was then placed in the reactor and deoxygenated by an argon flow of 30 mL/min applied for 30 min at 25 ^o^C. The oxygen concentration was monitored by the GC analysis. Once the system became free of oxygen, the illumination was applied followed by the GC analysis. All the visible light photocatalytic experiments were carried out at 40 ^o^C monitored by a thermocouple. The heat was supplied in order to maximize the hydrogen production allowing the detection of H_2_ gas in the case of samples with the lowest activity.

## Additional Information

**How to cite this article**: Nasalevich, M. A. *et al*. Electronic origins of photocatalytic activity in *d^0^* metal organic frameworks. *Sci. Rep.* **6**, 23676; doi: 10.1038/srep23676 (2016).

## Supplementary Material

Supplementary Information

## Figures and Tables

**Table 1:** Catalytic performance.

Sample	HOCO-LUCO gap/eV	Hydrogen evolution rate/μmol·h^−1^·g_catalyst_	Hydrogen evolution rate/μmol·h^−1^·mol(M^4+^)^−1^	External quantum efficiency/%
Ti	2.75	49.3	12747	0.022
Zr_i_	2.92	1	357^a^	0.0009
Hf_i_	2.92	1	467^a^	0.0009
Zr_d_	2.95	1.7	472^b^	0.0016
Hf_d_	2.97	1.7	654^b^	0.0016

^a^Stoichiometric crystal composition: M^4+^:ATA = 1:1.

^b^Defective crystals: M^4+^:ATA = 3:2.

**Table 2:** Fitting parameters of the transient absorption spectra for the MOF-catalysts.

Sample	Lifetime of transient signal/ps		
Ti	1.6	80	9·10^3^
Hf_d_	2.4	20	190
Zr_d_	1.6	10	130
Hf_i_	1.4	–	–
Zr_i_	1.5	–	–
