# Supplementary material for: Electronic origins of photocatalytic activity in d0 metal organic frameworks
Source: Sci Rep. 2016 Mar 29;6:23676. doi: 10.1038/srep23676 (PMC4810359; doi:10.1038/srep23676)
Supplement: Supplementary Information [file srep23676-s1.pdf]

Supplementary information to:

Electronic origins of photocatalytic activity in d^0 metal organic frameworks

Maxim A. Nasalevich,[a] Christopher H. Hendon,[b] Jara G. Santaclara,[a] Katrine Svane,[b] Bart van der Linden,[a] Sergey L. Veber,[c,d] Matvey V. Fedin,[c,d] Arjan J. Houtepen[e], Monique A. van der Veen,[a] Freek Kapteijn,[a] Aron Walsh,[b,f] and Jorge Gascon.[a,*]

[a]Catalysis Engineering, Department of Chemical Engineering, Delft University of Technology, Julianalaan 136, Delft, The Netherlands.
[b]Department of Chemistry, University of Bath, Claverton Down, Bath BA2 7AY, U. K.
[c]Laboratory of Magnetic Resonance, International Tomography Center, Institutskaya 3A, Novosibirsk 630090, Russia.
[d]Novosibirsk State University, Novosibirsk 630090, Russia.
[e]Optoelectronic Materials, Department of Chemical Engineering, Delft University of Technology, Julianalaan 136, 2628 BL Delft, The Netherlands
[f]Department of Materials Science and Engineering, Yonsei University, Seoul, Korea.

j.gascon@tudelft.nl

Table of contents:

1. Photocatalytic setup 3
2. Textural properties 4
3. Powder X-Ray diffraction 5
4. Scanning Electron Microscopy 6
5. Thermo Gravimetric Analysis (TGA) 7
6. Structural defects within the NH_2-UiO-66 frameworks 9
7. Tauc plot 11
8. EPR light source 12
9. External quantum efficiency (EQE) 13
10. Transient absorption spectroscopy 15
11. References 25

1. Photocatalytic setup

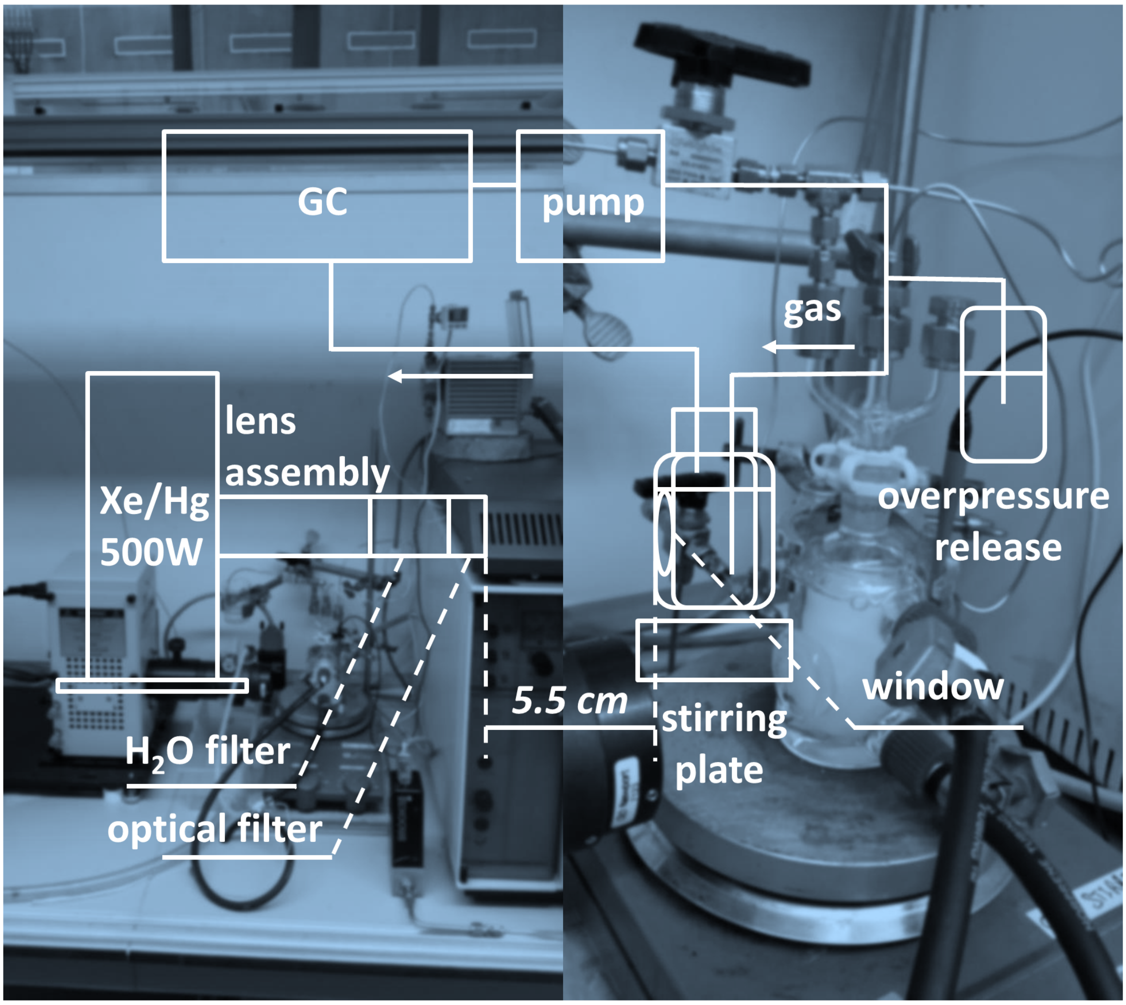

Figure S1. Schematic representation of the photocatalytic setup employed in this work.

2. Textural properties

N_2-physisorption experiments were carried out at 77 K in a TriStar II unit gas adsorption analyser (Micromeritics). Prior to the measurements the samples were degassed at 423 K under vacuum for 16 h. The BET areas were calculated using intervals allowing positive BET constants.[1] The total pore volumes were calculated at 0.9 relative pressure.

Table S1. Textural properties of the MOFs employed in this work.

Notation	MOF	S_{BET} / m^2g^{-1}	V_p / cm^3g^{-1}	Comment
Ti	NH_2-MIL-125(Ti)	1500	0.61	
Zr_i	NH_2-UiO-66(Zr)	959/700[a]/830[d]	0.41	'ideal crystals'
Hf_i	NH_2-UiO-66(Hf)	706	0.31	'ideal crystals'
Zr_d	NH_2-UiO-66(Zr)	1285/1150[b]/1200[c]	0.54	'defective crystals'
Hf_d	NH_2-UiO-66(Hf)	789	0.35	'defective crystals'

a: calculated BET area for the stoichiometric crystal (12 linkers per node)
b: calculated BET area for the defective crystal (8 linkers per node) both adopted from Farha et al.[2]
c: experimental BET area reported by Farha et al.[2]
d: experimental BET area reported by Farha et al. for the HCl-free synthesis[2]

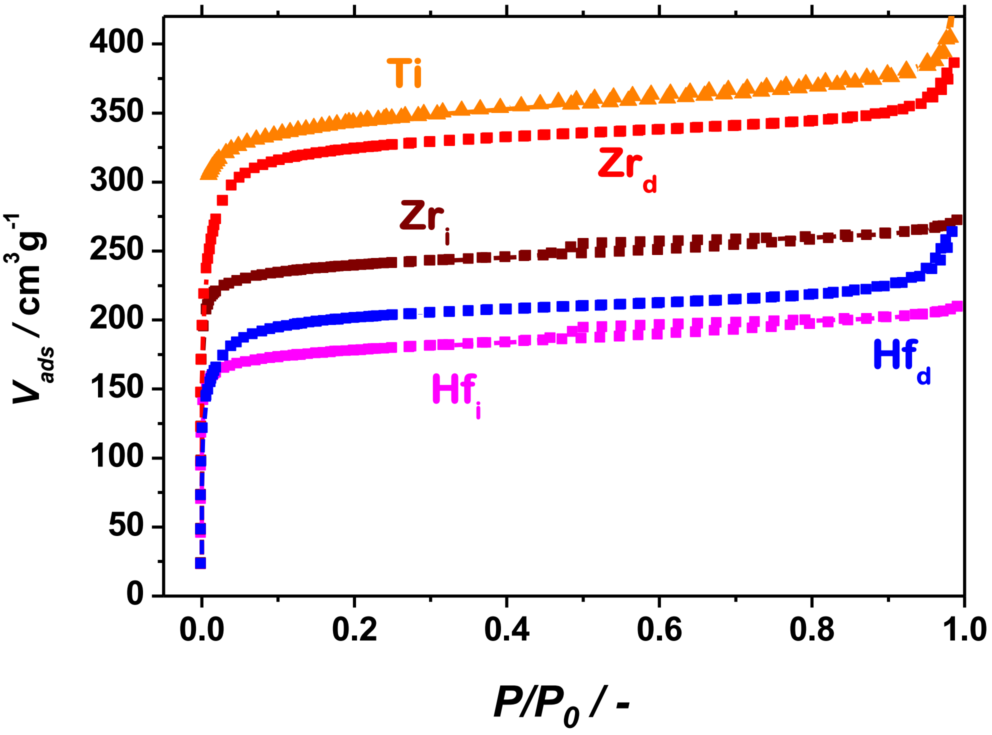

Figure S2. Nitrogen physisorption isotherms for the catalysts used in this work. Top to bottom: NH_2-MIL-125(Ti) (*orange*), defective NH_2-UiO-66(Zr) (*red*), ideal NH_2-UiO-66(Zr) (*bordeaux*), defective NH_2-UiO-66(Hf) (*blue*), ideal NH_2-UiO-66(Hf) (*magenta*).

3. Powder X-Ray diffraction

Powder X-Ray diffraction patterns were recorded using Bruker-AXS D5005 with Co-Kα radiation.

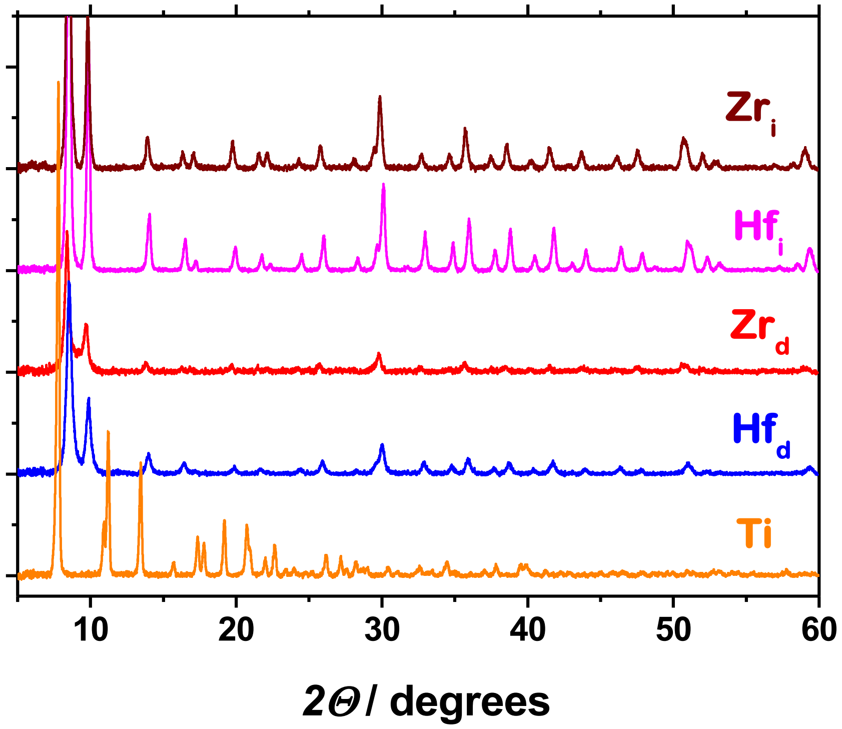

Figure S3.Powder X-Ray diffraction patterns of the catalysts used in this work: ideal NH_2-UiO-66(Zr) (*bordeaux*), ideal NH_2-UiO-66(Hf) (*magenta*), defective NH_2-UiO-66(Zr) (*red*), defective NH_2-UiO-66(Hf) (*blue*), NH_2-MIL-125(Ti) (*orange*).

4. Scanning Electron Microscopy

Scanning electron microscopy (SEM). Scanning electron microscopy (SEM) was carried out using a JEOL JSM-6010LA InTouchScope microscope.

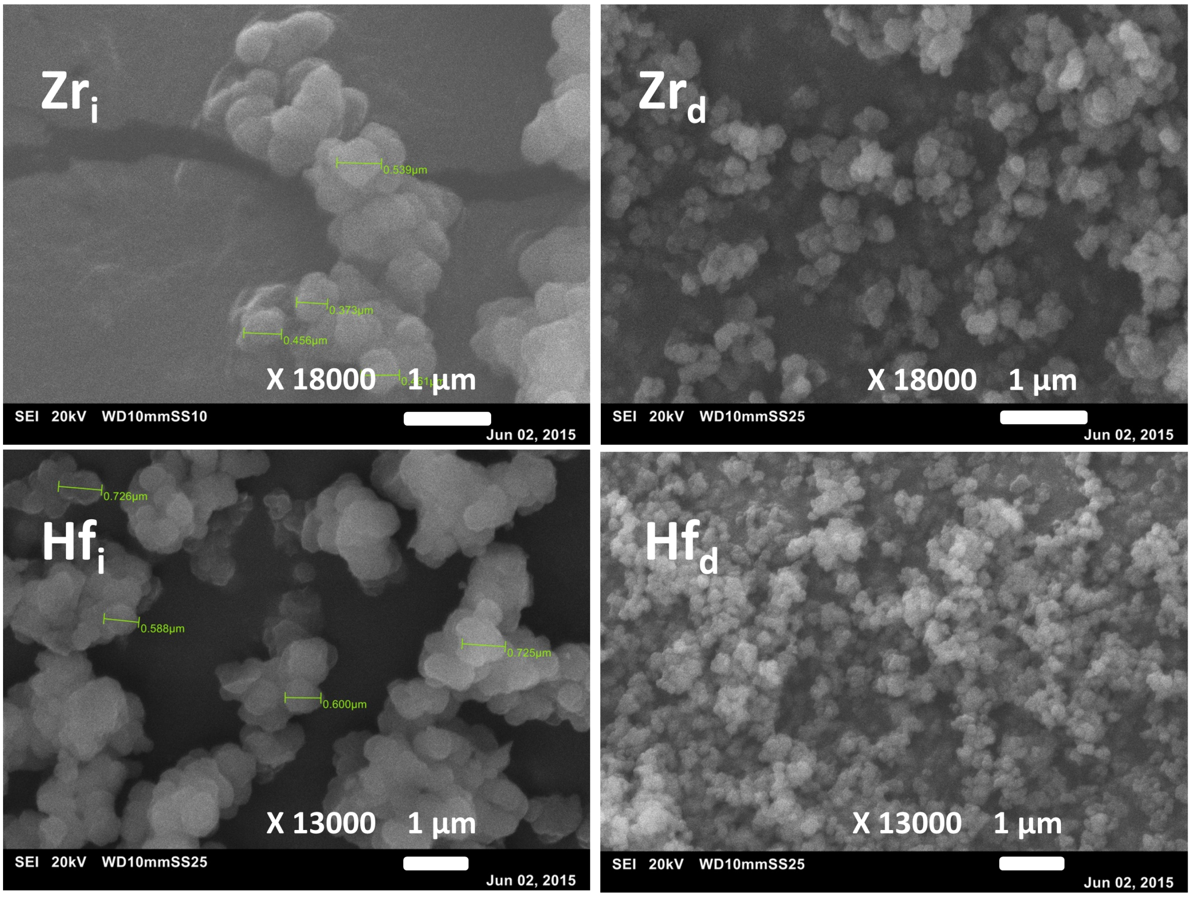

Figure S4. SEM micrographs of the UiO solids utilized in this work: ideal NH_2-UiO-66(Zr) (*top left*), defective NH_2-UiO-66(Zr) (*top right*), ideal NH_2-UiO-66(Hf) (*bottom left*), defective NH_2-UiO-66(Hf) (*bottom right*). The micrographs recorded at 18000 magnification (*top*) and 13000 (*bottom*).

5. Thermo Gravimetric Analysis (TGA)

Thermogravimetric analysis was performed by means of Mettler Toledo TGA/SDTA851e, under an air flow of 60 ml min^{-1} at heating rates of 10 K min^{-1} up to 1073 K.

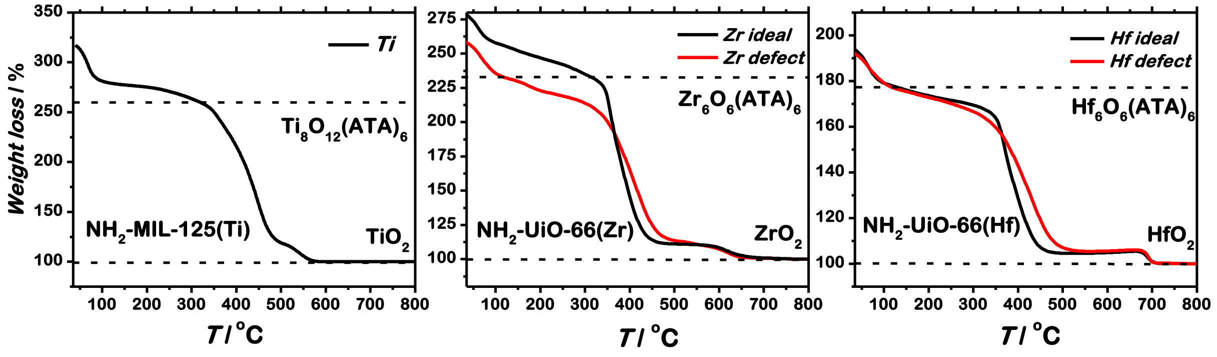

Figure S5. TGA profiles of NH$_2$-MIL-125(Ti) (*left*), NH$_2$-UiO-66(Zr) (*centre*) and NH$_2$-UiO-66(Hf) (*right*). The analysis is carried out in air. The profiles were normalized with respect to the remaining oxide (corresponds to 100% weight loss). The dashed lines represent the anhydrous MOF (*top*) and the remaining MO$_2$ (*bottom*).

To describe the thermal decomposition/oxidation in air of the MOFs used in this study we used the following equations:

$$2Ti_8O_{36}C_{48}H_{34}N_6 + 105O_2 \rightarrow 16TiO_2 + 96CO_2 + 34H_2O + 12NO_2 \quad \text{(s1)}$$

$$2Zr_6O_{30}C_{48}H_{30}N_6 + 105O_2 \rightarrow 12ZrO_2 + 96CO_2 + 30H_2O + 12NO_2 \quad \text{(s2)}$$

$$2Hf_6O_{30}C_{48}H_{30}N_6 + 105O_2 \rightarrow 12HfO_2 + 96CO_2 + 30H_2O + 12NO_2 \quad \text{(s3)}$$

The weights of the materials after treatment at 750 ^{o}C were converted to 100%, representing the oxidic phase of the used metal. The dashed lines in Figure 1 of the main text represent the weight difference between the oxides and the anhydrous MOFs for the case of stoichiometric crystals. Such differences were calculated assuming that the combustion processes proceed via the reactions (s1)-(s3) and the only remaining solid products are ZrO$_2$, HfO$_2$ and TiO$_2$. In this case the nitrogen is accounted for in the form of NO$_2$. An example of such calculation is given below:

$$Zr_6O_6 - (O_2C - C_6H_3NH_2 - CO_2)_6$$

According to (2), $\nu_{MOF} : \nu_{ZrO_2} = 1:6$

$M_{MOF} = 1718.12\ \text{g} \cdot \text{mol}^{-1}$ $\qquad$ $M_{ZrO_2} = 123.22\ \text{g} \cdot \text{mol}^{-1}$

For the case of a stoichiometric crystal

$$\frac{m_{MOF}}{m_{ZrO_2}} = \frac{\nu_{MOF} \cdot M_{MOF}}{\nu_{ZrO_2} \cdot M_{ZrO_2}} = \frac{1 \cdot 1718.12}{6 \cdot 123.22} = 2.32 = 232\% \qquad \text{(s4)}$$

This means that if the mass of the remaining ZrO_2 is assumed to be 100%, the anhydrous stoichiometric NH_2-UiO-66(Zr) must be positioned at 232% of weight loss scale. The same reasoning was used for calculating the expected weight losses for Ti and Hf.

6. Structural defects within the NH_2-UiO-66 frameworks

The UiO-type materials are particularly known for possessing structural defects, as demonstrated by the non-stoichiometric metal to linker ratios observed by several groups experimentally.The missing linkers are often seen being compensated by formates,[3] oxygen, hydroxyl groups, water[2] and/or chloride,[4] however a comprehensive structural information is missing. The influence of these structural defects on gas sorption was documented by Snurr et al.[5]and Zhou et al.[6] In order to consider the influence of the defects on the electronic and photocatalytic properties of NH_2-UiO-66(X) materials, two synthetic protocols were followed in this work. The first method yields highly defective structures, as documented by Farha and co-workers, whereas the second approach was claimed to produce nearly stoichiometric UiO crystals. These two types of crystals are denoted X_d and X_i (X = Zr or Hf) and referred to as 'defective' and 'ideal' respectively. Well in line with the previous reports, the low-temperature protocol results in a defective structure as can be concluded from the BET area exceeding the theoretical prediction for NH_2-UiO-66(Zr) (see Table S1). The BET area is slightly higher than the predicted value for the case of 8 linkers per node instead of the stoichiometric 12 (1285 vs. 1150 m^2/g). The structural defects in Zr_d are also reflected by the poor crystallinity of the solid (Figure S3) as well as the small particle size as is evident from the broadening of the Bragg reflections. Scanning electron microscopy confirms these observations as depicted in Figure S4. At the same time the high-temperature synthetic route yields NH_2-UiO-66(Zr) material, Zr_i, possessing high crystallinity, better-distinguishable crystal shape and the surface area closely matching the predicted value for an ideal structure. The observations for the case of NH_2-UiO-66(Hf) are similar, yet less prominent. The crystallinity of Hf_d is heavily affected by the synthesis procedure. However, the difference in textural properties of Hf_d and Hf_i is less dramatic than in the case of Zr-based MOFs.

Additional investigation of the defects within the UiO MOFs was carried out using thermogravimetric analysis (TGA). The TGA profiles are depicted in Figure S5. Similarly to the report of Lillerud*et al.*,[4] TGA profiles of the catalysts utilized in this work were normalized with respect to the remaining corresponding oxides. While no clear plateau was observed for UiO-66(Zr) derivatives including NH_2-UiO-66(Zr)[2], our experiments allowed for a quantitative TGA analysis. The first weight loss observed up to 110 ^oC is associated with the solvent removal from the pores of the framework. The second step is the gradual dehydroxylation of the framework, giving rise to $Zr_6O_6(ATA)_6$ followed by the drop at around 400 ^oC that corresponds to the destruction of the MOF. The decomposition of the ideal sample

starts at the predicted weigh loss value of 232% with respect to the pure oxide (Figure S5 centre), whereas in the defective MOF the amount of organics is lower. In view of these results, we can conclude that the low temperature synthesis of NH_2-UiO-66(Hf) does not yield MOF crystals completely free of defects, yet the plateau of the ideal sample is higher, in line with N_2 physisorption results. The presence of structural defects is also typical for Hf-based frameworks of the UiO family.[7,8] The weight of NH_2-MIL-125(Ti) sample before the full destruction of the framework begins (*ca.* 350 ^{o}C) corresponds well to the theoretical expectations thus suggesting nearly ideal structure of the MOF.

7. Tauc plot

Diffuse reflectance UV/Vis spectra were collected using a Perkin–Elmer Lambda 900 spectrophotometer equipped with an integrating sphere (‘‘Labsphere”) in the 200–800 nm range. $BaSO_4$ was used as a white standard. The *Tauc* plot given below was obtained by plotting $F^2(R)\cdot(h\nu)^2$, where $F(R) = (1 - R)^2/2R$, R is the measured reflectance, as a function of energy of light.

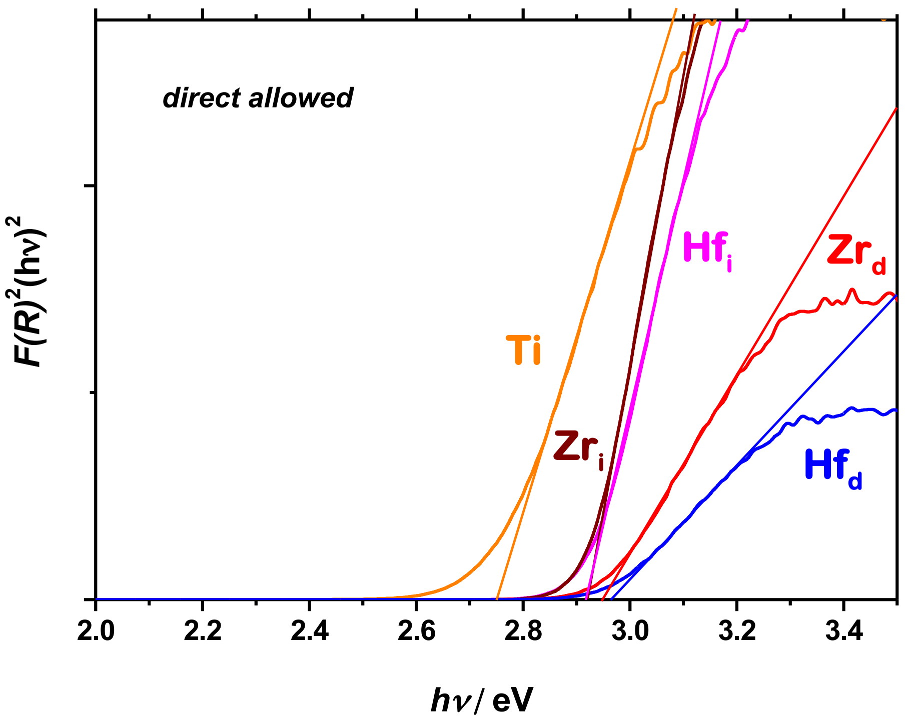

Figure S6. Tauc plot for the MOFs utilized in this work: NH_2-MIL-125(Ti) (*orange*), defective NH_2-UiO-66(Zr) (*red*), ideal NH_2-UiO-66(Zr) (*bordeaux*), defective NH_2-UiO-66(Hf) (*blue*), ideal NH_2-UiO-66(Hf) (*magenta*). The curves are obtained under the allowed direct transition assumption.

Noteworthy, the absorption maxima of the ideal and defective samples are at nearly the same position, although there is a slight blue shift (*ca.* 7 nm) of the absorption onset in the case of Zr_d and Hf_d. Another difference when structural defects are present is the reduced intensity of the lowest energy absorption band when compared against the other transition (λ_{max} *ca.* 250 nm). Since this band is ascribed to the linker excitation, a different linker to metal ratio in the structure could account for the difference in relative intensities. More importantly, the optical spectra of the ideal Zr and Hf MOF pair and those of the defective pair are identical.

8. EPR light source

EPR spectroscopy. Steady-state EPR measurements were carried out at X-band (9.52 GHz) using a commercial EPR spectrometer Bruker Elexsys E580 equipped with an Oxford Instruments temperature control system (T = 4 - 300 K). All spectra were acquired at 40 K. Samples were prepared by suspending 25 mg of a MOF in a solution of electron donor triethylamine (TEA)/CH_3CN/H_2O = 1/5/0.1 (total volume 200 μL). The volume of suspension placed into the EPR resonator was 50-60 μL. Each sample was degassed by several freeze-pump-thaw procedures and then sealed in an EPR quartz tube (OD 3.8 mm, ID 2.8 mm). When needed, the samples were exposed to a 500 W mercury lamp equipped with an IR filter (H_2O, 7 cm optical path) and a UFS6 filter (spectrum given below) for 30 min. After this period the sample tube was placed into liquid nitrogen. The cool-down time to frozen state was ~ 10-15 s. Then it was inserted into EPR cryostat.

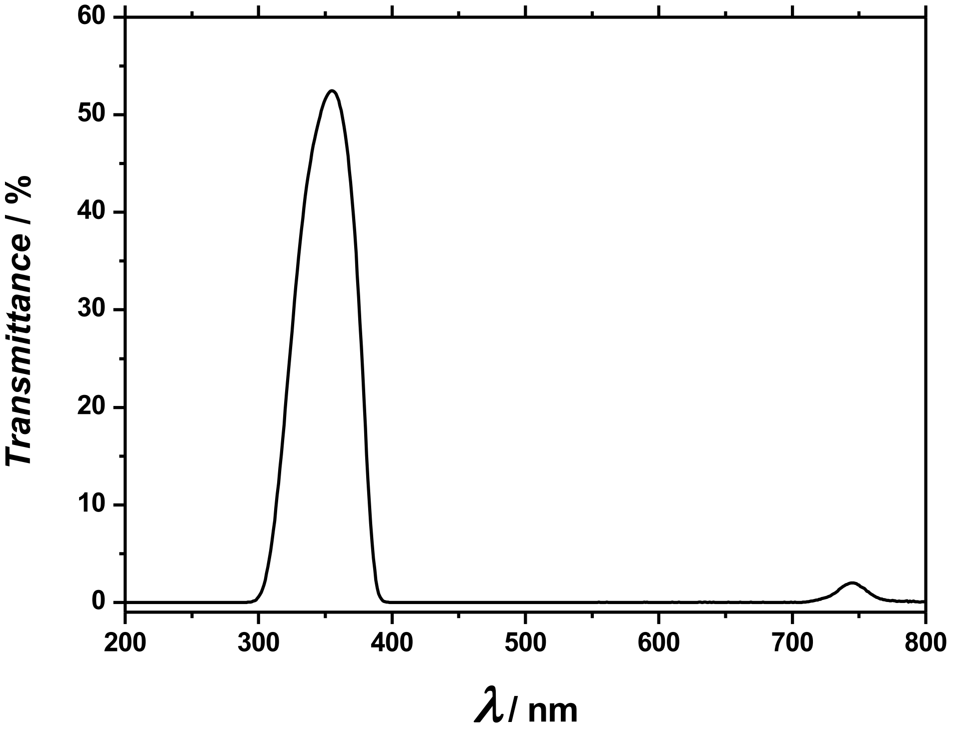

Figure S7. Transmittance of the UFS6 filter used in the CW EPR experiments.

9. External quantum efficiency (EQE)

External quantum efficiencies of catalysts employed in this work were calculated using the following equation:

$$\Phi = \frac{E(t)}{P(t)},$$

Where *E(t)* is the number of electrons carrying out a redox reaction per unit of time; *P(t)* is a photon flux per unit of time. The incident photon flux was determined with the help of the photon counter as specified in the experimental part to obtain:

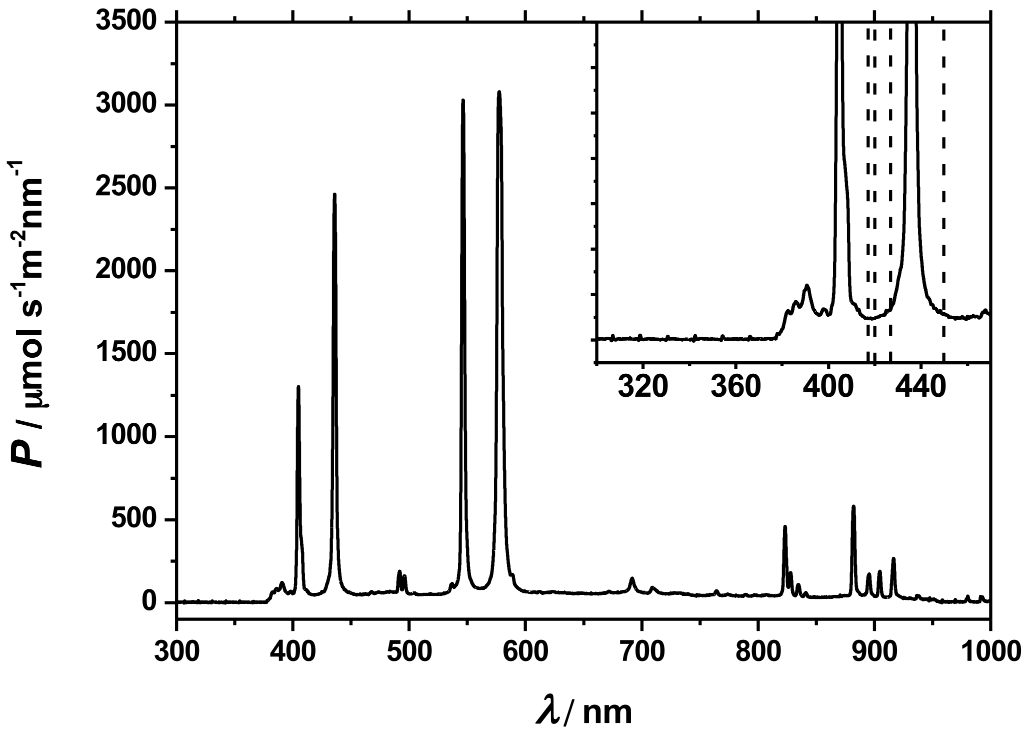

Figure S8. Incident photon flux as a function of wavelength of the 500 W Xe/Hg light source used in this work. The measurement is carried out at the distance of 5.5 cm with the liquid filter on. The inset highlights the spectral region relevant for the hydrogen evolution catalysed by the MOFs. The dashed lines denote cut-off wavelengths for each catalyst as determined from *Tauc* plot.

The spectrum was taken at the conditions of the photocatalytic reactions: 385 nm cut-off filter, liquid filter on, the distance 5.5 cm. Then the curve was integrated with the lower boundary being 350 nm, where the flux goes to 0. The upper boundary for the integration was determined by using the *Tauc* plot (Figure S6) and converting the HOCO-LUCO gaps back to nm using the relation λ = 1240/*E*(eV). The integration yielded the following photon fluxes:

16815, 6839, 6839, 6582 and 6438 μmol(photons)·$s^{-1}m^{-2}$. The spot size of the beam reaching the reactor window was 2.27 cm^2. Details of the calculation for the case of Ti are given below:

$$P_{Ti}(t) = \int_{350}^{451} P(t,\lambda)d\lambda \cdot 0.000227\,\mathrm{m}^2 = 16815\,\mu\mathrm{mol(photons)}\cdot\mathrm{s}^{-1}\mathrm{m}^{-2}\cdot 0.00027\,\mathrm{m}^2 =$$
$$= 3.817\,\mu\mathrm{mol(photons)}\cdot\mathrm{s}^{-1}$$

To account for the number of photons required to assemble one hydrogen molecule (2) the reaction rates must be multiplied by 2 and converted to appropriate units to obtain the number of electrons:

$$E(t) = r(H_2)\cdot 2 = 1.484\,\mu\mathrm{mol}\cdot\mathrm{h}^{-1}\cdot 2 = 2.968\,\mu\mathrm{mol}\cdot\mathrm{h}^{-1} = 8.244\cdot 10^{-4}\,\mu\mathrm{mol}\cdot\mathrm{s}^{-1}$$

$$\Phi_{Ti} = \frac{E(t)}{P(t)} = \frac{8.244\cdot 10^{-4}\,\mu\mathrm{mol}\cdot\mathrm{s}^{-1}}{3.817\,\mu\mathrm{mol}\cdot\mathrm{s}^{-1}} = 2.16\cdot 10^{-4} = 0.0216\,\%$$

All the other EQEs were calculated in the same manner.

10. Transient absorption spectroscopy

Samples were excited using 180 fs pulses at 400 nm for NH_2-MIL-125(Ti) and 370 nm for NH_2-UiO-66(Zr) and NH_2-UiO-66(Hf) generated in a YKGBW oscillator (Light Conversion, Pharos SP) at 1028 nm through nonlinear frequency mixing in an OPA and second harmonics module (Light Conversion, Orpheus). A small fraction of the 1028 nm fundamental beam was split off to generate the broadband probe spectrum in a sapphire (500 – 1600 nm) crystal. The probe pulse was delayed relative to the pump using a delay stage with maximum delay of 3 ns. The pump and probe pulses overlap on the sample position under an angle of ~8 degrees, after which the probe light is led to a detector suitable for the probe spectrum selected (Ultrafast Systems, Helios). In order to prevent multiple photons absorption processes, the pump fluence was set sufficiently low, allowing us to study single exciton dynamics. In a typical experiment 2.7 mg of a MOF were dispersed in acetonitrile (700 µL) and sonicated for 30 min. In order to separate large particles (> 100 nm), the suspension was then centrifuged for 8 min at 10000 rpm. The supernatant was placed in a 2 mm stirred quartz cuvette for the measurements. Transient data were analyzed using a global fitting routine in which the spectral evolution of the time-dependent absorption difference spectra is fitted to a **sequential** model yielding evolution-associated difference spectra (EADS).[9] In the case of the NH_2-MIL-125(Ti) and NH_2-UiO-66(Zr/Hf) 'defective' a three-state kinetic model was used to fit the experimental data.[10] An additional state was used to force the system to go to the ground state. All time traces were satisfactorily fitted with three time constants of which the last state decays to the ground state being part of the model. For the NH_2-UiO-66(Zr/Hf) 'ideal' one excited state (which population's decay is defined by $exp(-k\tau)$) that goes to the ground state (described by $1-exp(-k\tau)$) was sufficient for a good fit.

When only single photon absorption processes occur, a linear correlation between the pump power and the change in absorption should be registered. In order to determine whether multiple photons absorption processes are being observed, a region of the data is selected for every measurement. This region contains the data at the highest intensity; it is averaged for each pump power and then fitted with a linear fit. The resulting relations are referred to as linearity checks and are given below alongside the evolution-associated difference spectra (EADS) and characteristic decays of photoexcited states.

- **NH_2-MIL-125(Ti)**

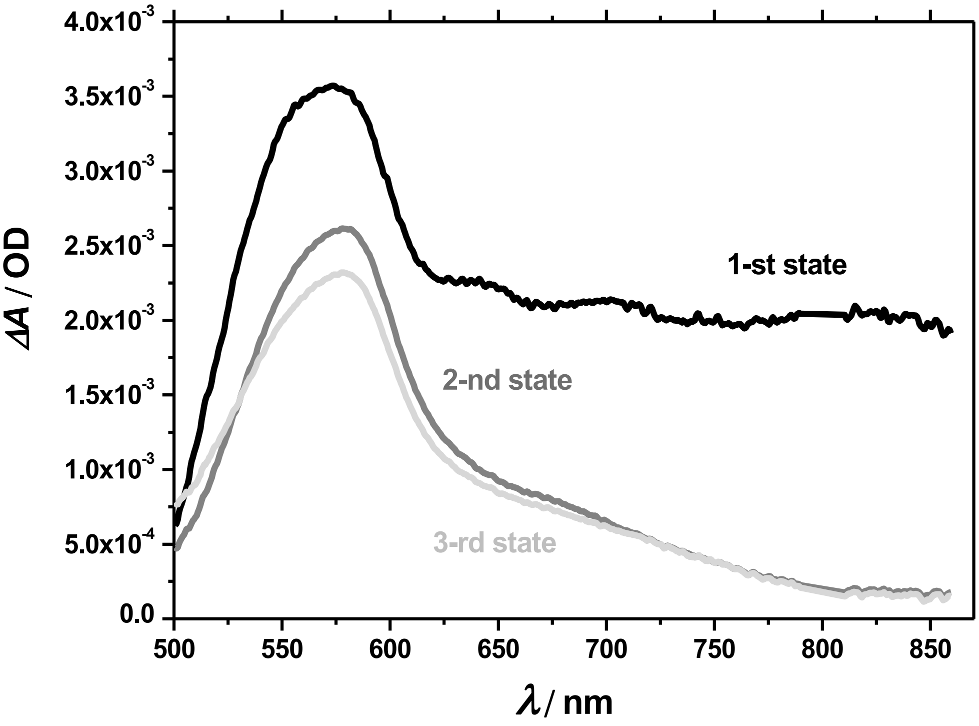

Figure S9. Evolution-associated difference spectra of NH_2-MIL-125(Ti) upon 400 nm excitation. The kinetic model exploits three transient consecutive states followed by a forced decay to the ground state.

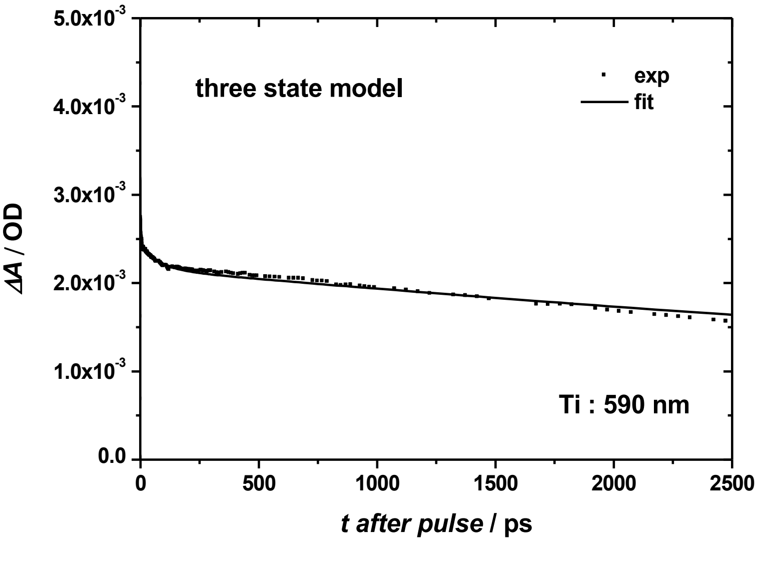

Figure S10. Representative time traces of NH_2-MIL-125 recorded at 590 nm upon excitation at 400 nm with excitation power of 10.1 mW: experimental data (*squares*) and fitting (*black line*).The kinetic model exploits three transient consecutive states followed by a decay to the ground state.

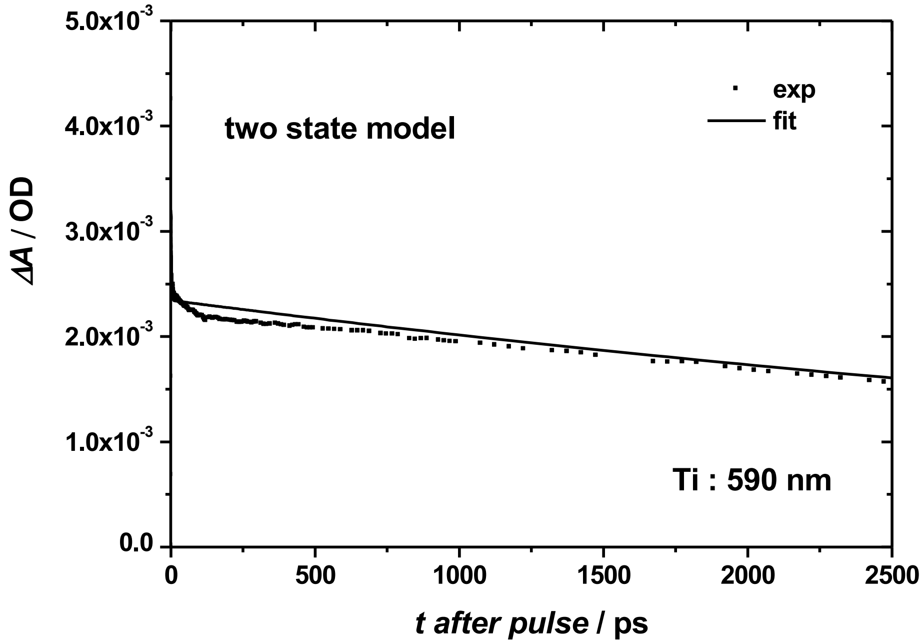

Figure S11. Representative time traces of NH_2-MIL-125(Ti) recorded at 590 nm upon excitation at 400 nm with excitation power of 10.1 mW: experimental data (*squares*) and fitting (*black line*). The fit is obtained by using a two-state model that is forced to decay to the ground state. The figure highlights the necessity of employing the three state model.

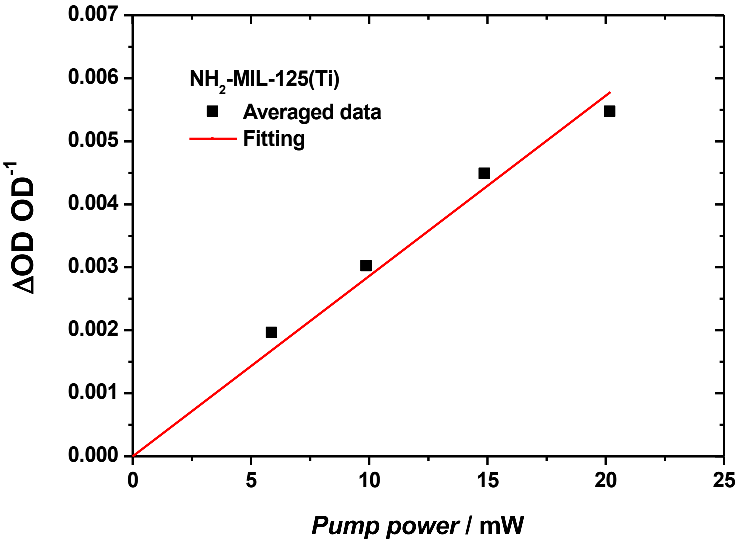

Figure S12. Linearity check for NH_2-MIL-125(Ti).

- **NH_2-UiO-66(Zr) 'ideal'**

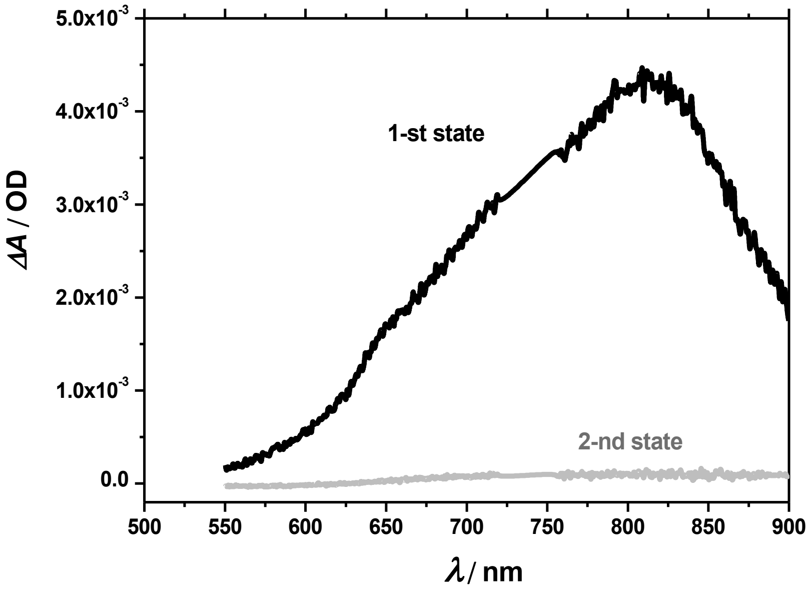

Figure S13. Evolution-associated difference spectra of NH_2-UiO-66(Zr) 'ideal' upon 370 nm excitation.The kinetic model exploits two transient consecutive states. As the 2-nd transient state is practically 0, it was assumed to be the ground state, no extra constraints added.

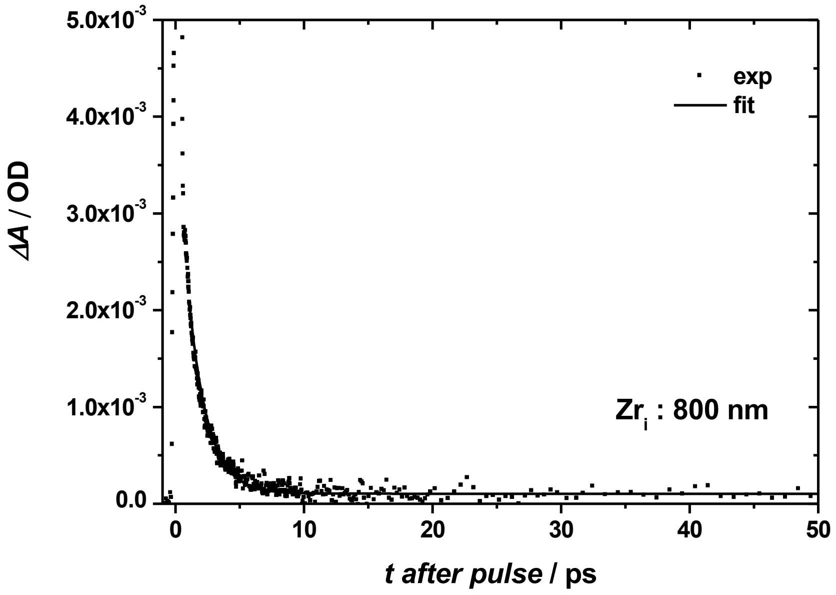

Figure S14. Representative time traces of NH_2-UiO-66(Zr) 'ideal' recorded at 800 nm upon excitation at 370 nm with excitation power of 26.9 mW: experimental data (*squares*) and fitting (*black line*).The kinetic model exploits two transient consecutive states. As the 2-nd transient state is practically 0, it was assumed to be the ground state, no extra constraints added.

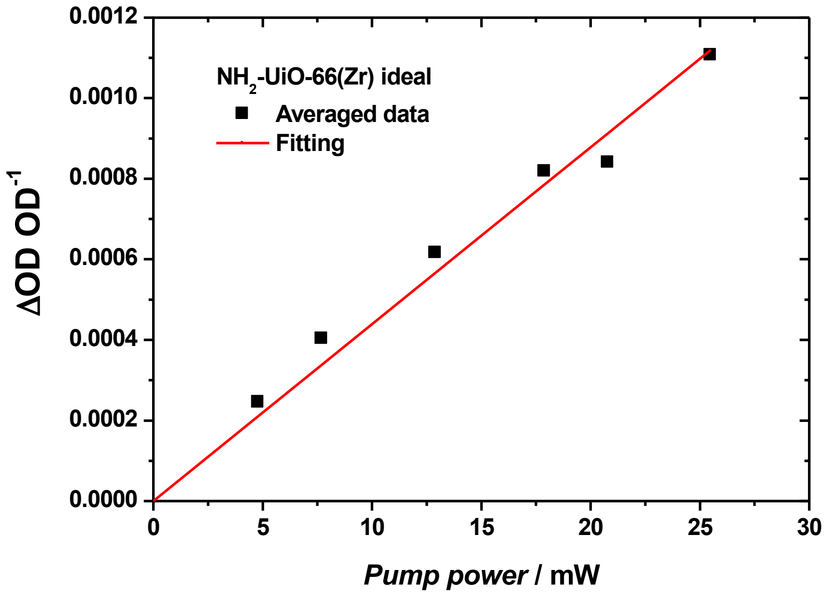

Figure S15. Linearity check for NH_2-UiO-66(Zr) 'ideal'.

- **NH_2-UiO-66(Hf) 'ideal'**

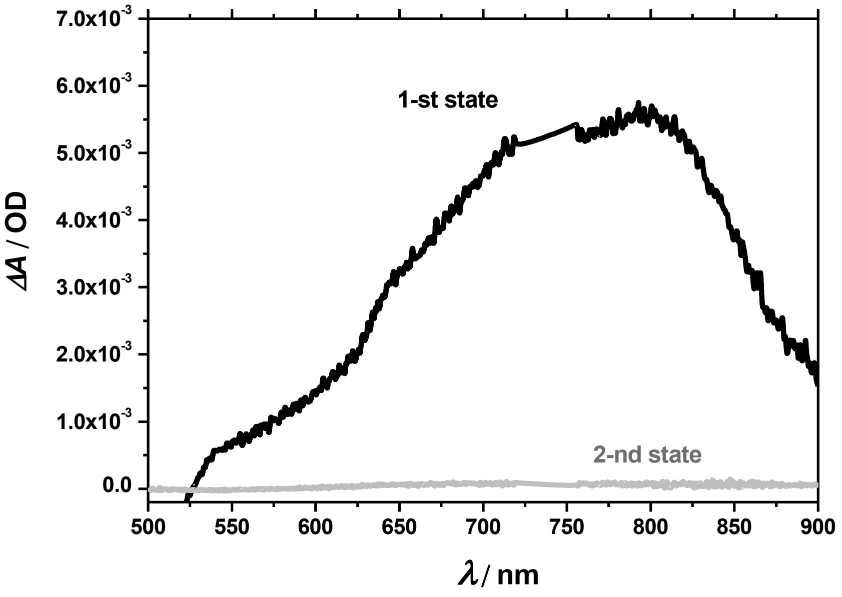

Figure S16. Evolution-associated difference spectra of NH_2-UiO-66(Hf) 'ideal' upon 370 nm excitation.The kinetic model exploits two transient consecutive states. As the 2-nd transient state is practically 0, it was assumed to be the ground state, no extra constraints added.

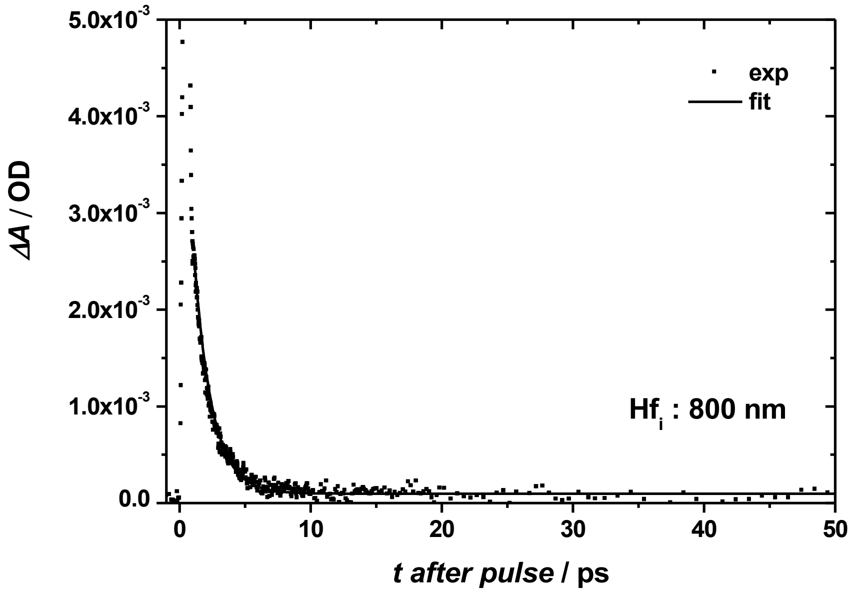

Figure S17. Representative time traces of NH_2-UiO-66(Hf) 'ideal' recorded at 800 nm upon excitation at 370 nm with excitation power of 26.3 mW: experimental data (*squares*) and fitting (*black line*).The kinetic model exploits two transient consecutive states. As the 2-nd transient state is practically 0, it was assumed to be the ground state, no extra constraints added.

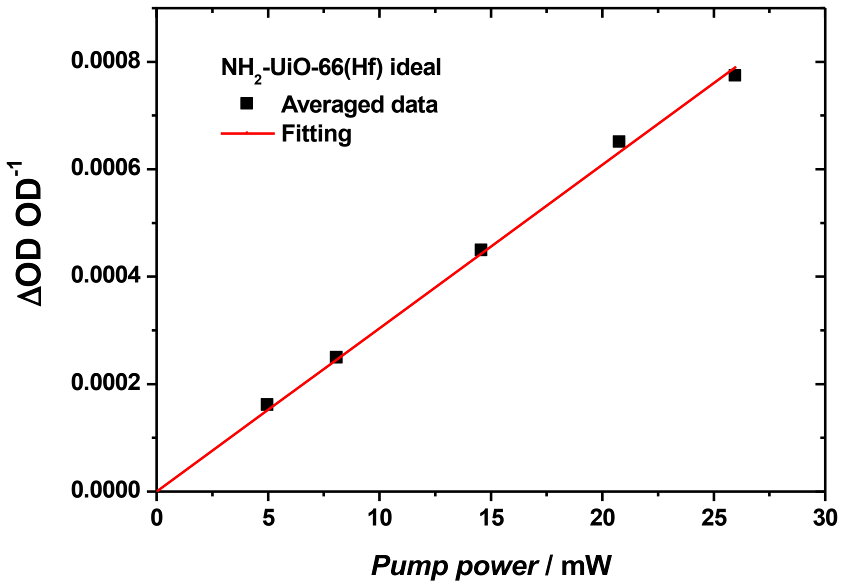

Figure S18.Linearity check for NH_2-UiO-66(Hf) 'ideal'.

- **NH_2-UiO-66(Zr) ‘defective’**

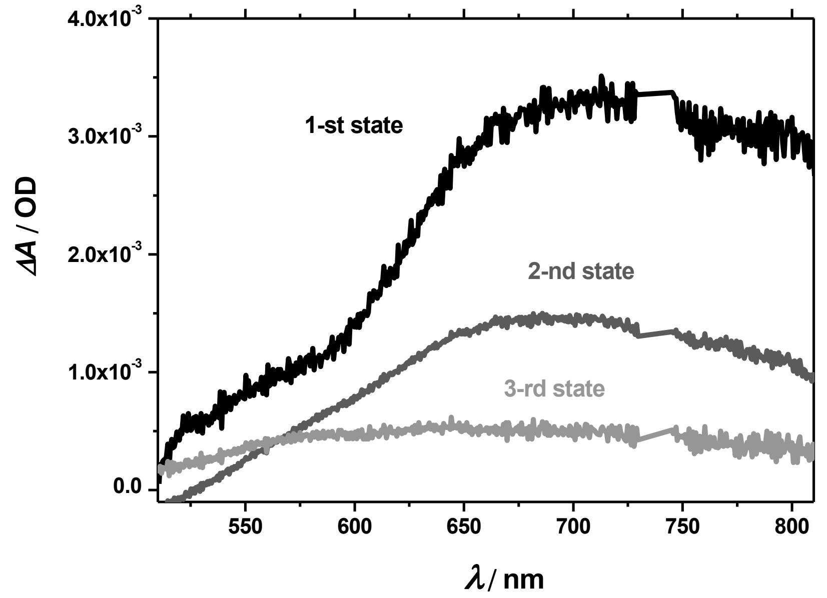

Figure S19. Evolution-associated difference spectra of NH_2-UiO-66(Zr) ‘defective’ upon 370 nm excitation.The kinetic model exploits three transient consecutive states followed by a forced decay to the ground state.

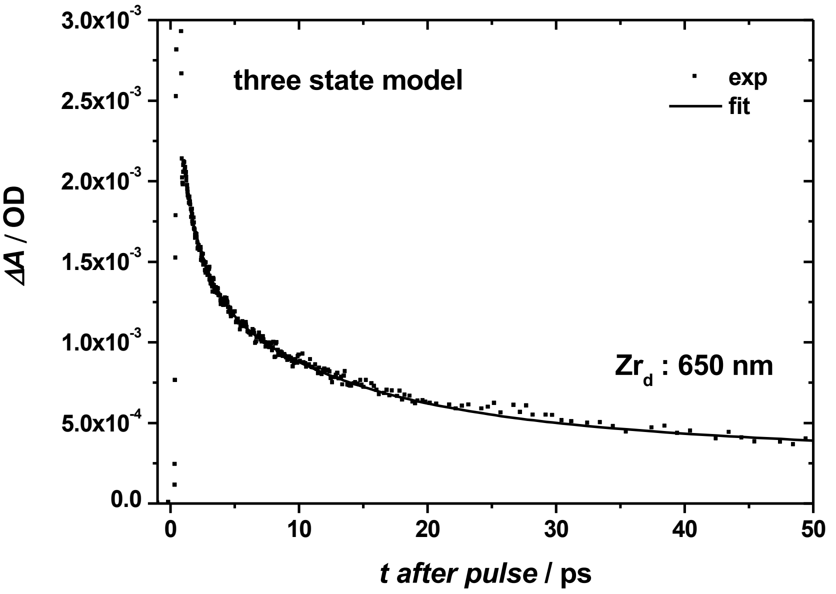

Figure S20. Representative time traces of NH_2-UiO-66(Zr) ‘defective’ recorded at 650 nm upon excitation at 370 nm with excitation power of 10.8 mW: experimental data (*squares*) and fitting (*black line*).The kinetic model exploits three transient consecutive states followed by a forced decay to the ground state.

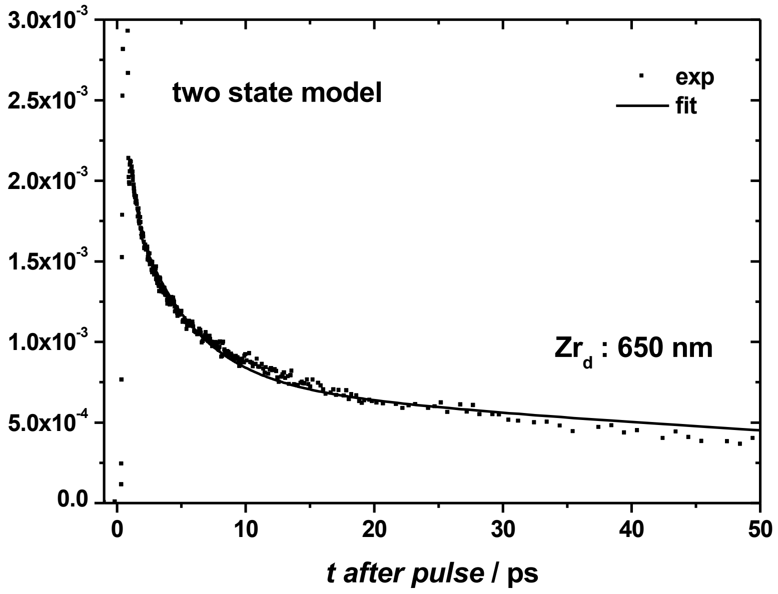

Figure S21.Representative time traces of NH_2-UiO-66(Zr) ‘defective’ recorded at 650 nm upon excitation at 370 nm with excitation power of 10.8 mW: experimental data (*squares*) and fitting (*black line*). The kinetic model exploits two transient consecutive states followed by a forced decay to the ground state. The figure highlights the necessity of employing the three state model.

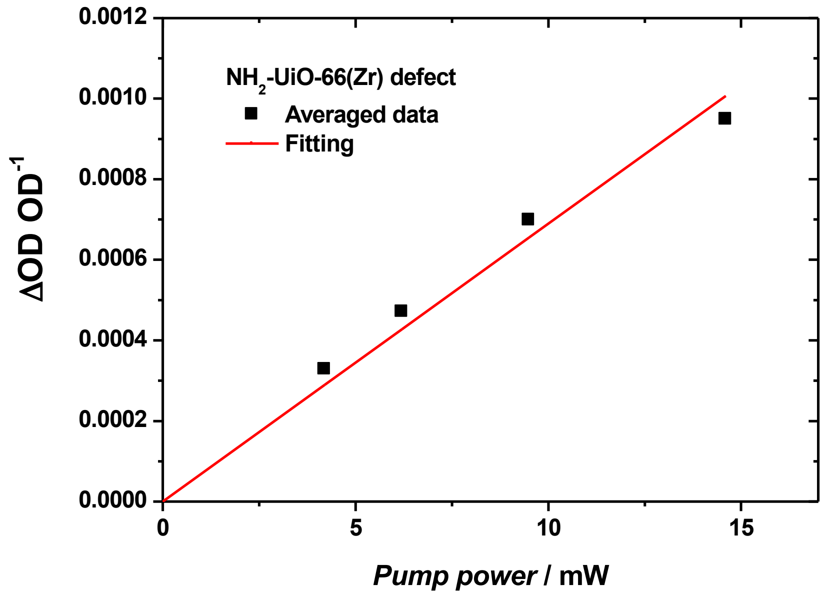

Figure S22.Linearity check for NH_2-UiO-66(Zr) ‘defective’.

- **NH_2-UiO-66(Hf) 'defective'**

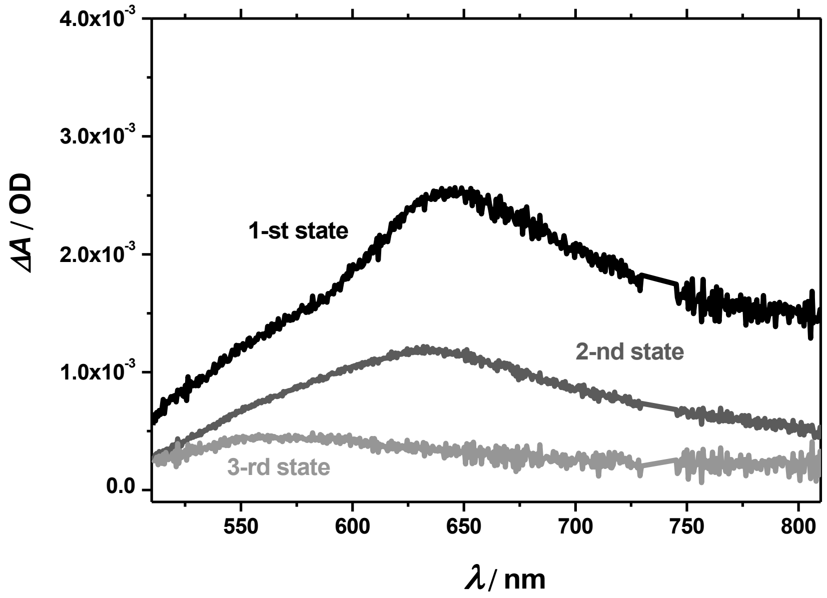

Figure S23. Evolution-associated difference spectra of NH_2-UiO-66(Hf) 'defective' upon 370 nm excitation.The kinetic model exploits three transient consecutive states followed by a forced decay to the ground state.

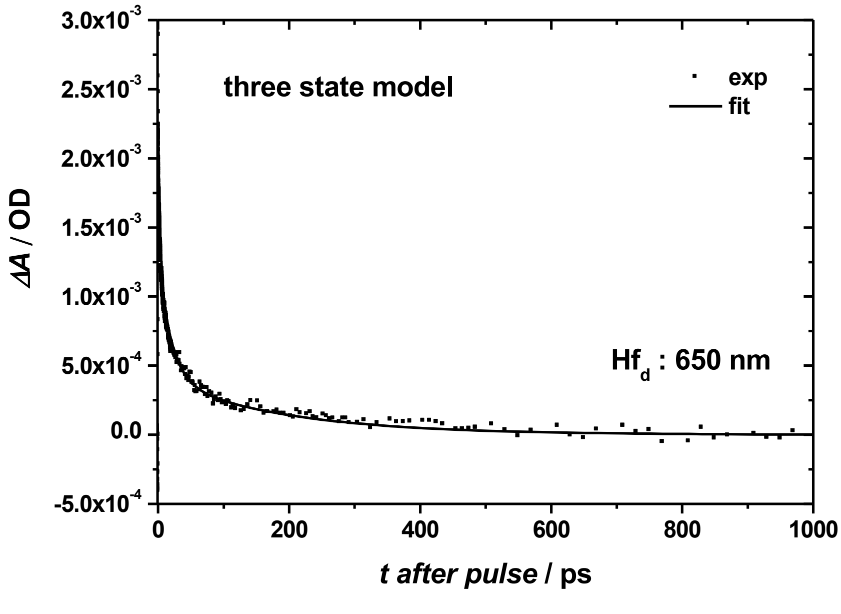

Figure S24. Representative time traces of NH_2-UiO-66(Hf) 'defective' recorded at 650 nm upon excitation at 370 nm with excitation power of 10.8 mW: experimental data (*squares*) and fitting (*black line*).The kinetic model exploits three transient consecutive states followed by a forced decay to the ground state.

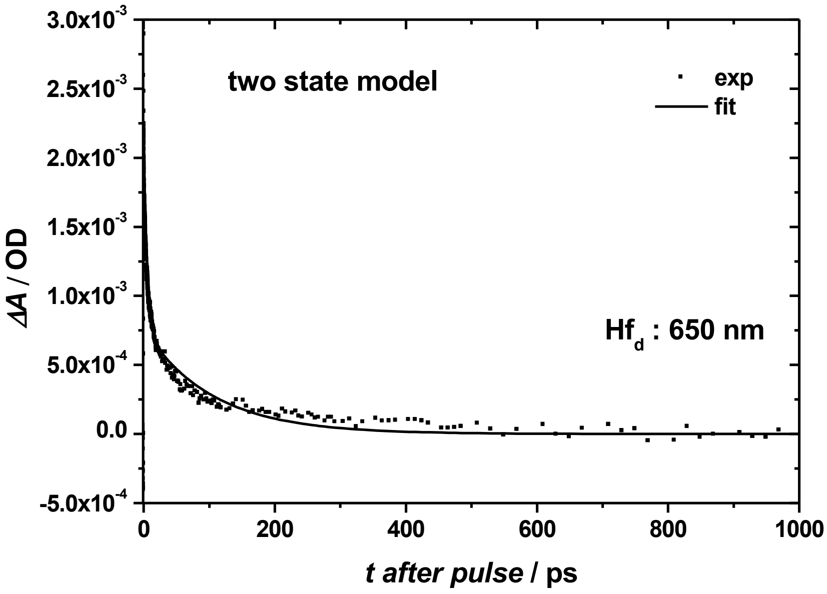

Figure S25. Representative time traces of NH_2-UiO-66(Hf) 'defective' recorded at 650 nm upon excitation at 370 nm with excitation power of 10.8 mW: experimental data (*squares*) and fitting (*black line*). The kinetic model exploits two transient consecutive states followed by a forced decay to the ground state. The figure highlights the necessity of employing the three state model.

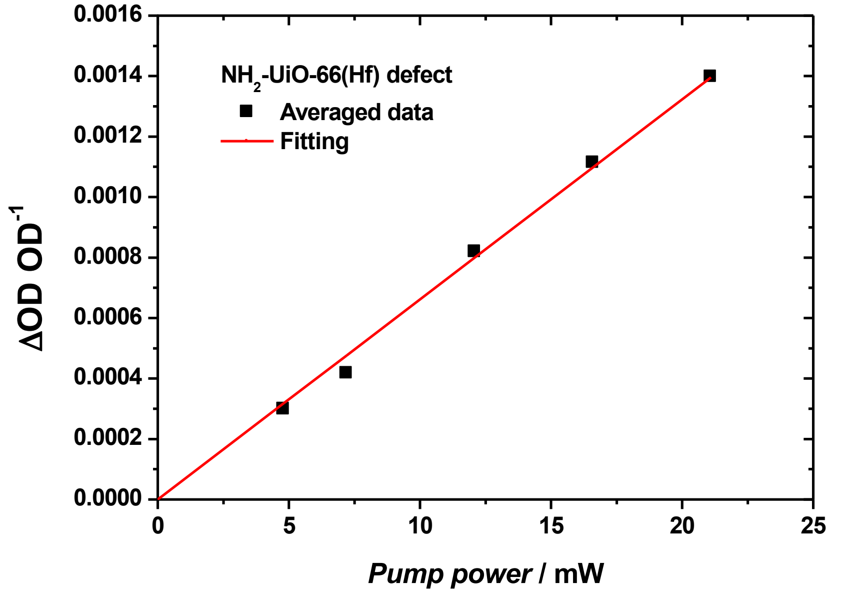

Figure S26. Linearity check for NH_2-UiO-66(Hf) 'defective'

11.References

(1) De Lange, M. F.; Vlugt, T. J. H.; Gascon, J.; Kapteijn, F. *Microporous and Mesoporous Materials* **2014**, *200*, 199.
(2) Katz, M. J.; Brown, Z. J.; Colon, Y. J.; Siu, P. W.; Scheidt, K. A.; Snurr, R. Q.; Hupp, J. T.; Farha, O. K. *Chemical Communications* **2013**, *49*, 9449.
(3) Furukawa, H.; Gándara, F.; Zhang, Y.-B.; Jiang, J.; Queen, W. L.; Hudson, M. R.; Yaghi, O. M. *Journal of the American Chemical Society* **2014**, *136*, 4369.
(4) Shearer, G. C.; Chavan, S.; Ethiraj, J.; Vitillo, J. G.; Svelle, S.; Olsbye, U.; Lamberti, C.; Bordiga, S.; Lillerud, K. P. *Chemistry of Materials* **2014**, *26*, 4068.
(5) Ghosh, P.; Colon, Y. J.; Snurr, R. Q. *Chemical Communications* **2014**, *50*, 11329.
(6) Wu, H.; Chua, Y. S.; Krungleviciute, V.; Tyagi, M.; Chen, P.; Yildirim, T.; Zhou, W. *Journal of the American Chemical Society* **2013**, *135*, 10525.
(7) Cliffe, M. J.; Wan, W.; Zou, X.; Chater, P. A.; Kleppe, A. K.; Tucker, M. G.; Wilhelm, H.; Funnell, N. P.; Coudert, F.-X.; Goodwin, A. L. *Nat Commun* **2014**, *5*.
(8) Cliffe, M. J.; Hill, J. A.; Murray, C. A.; Coudert, F.-X.; Goodwin, A. L. *Physical Chemistry Chemical Physics* **2015**, *17*, 11586.
(9) van Stokkum, I. H. M.; Larsen, D. S.; van Grondelle, R. *Biochimica et Biophysica Acta (BBA) - Bioenergetics* **2004**, *1658*, 262.
(10) Hunger, J.; Sonnleitner, T.; Liu, L.; Buchner, R.; Bonn, M.; Bakker, H. J. *The Journal of Physical Chemistry Letters* **2012**, *3*, 3034.
